# The Gut Microbiome in Parkinson’s Disease: A Longitudinal Study of the Impacts on Disease Progression and the Use of Device-Assisted Therapies

**DOI:** 10.3389/fnagi.2022.875261

**Published:** 2022-05-17

**Authors:** Michal Lubomski, Xiangnan Xu, Andrew J. Holmes, Samuel Muller, Jean Y. H. Yang, Ryan L. Davis, Carolyn M. Sue

**Affiliations:** ^1^Department of Neurology, Royal North Shore Hospital, St Leonards, NSW, Australia; ^2^Department of Neurogenetics, Kolling Institute, Faculty of Medicine and Health, University of Sydney, St Leonards, NSW, Australia; ^3^School of Medicine, The University of Notre Dame Australia, Sydney, NSW, Australia; ^4^School of Mathematics and Statistics, Sydney Precision Bioinformatics, University of Sydney, Camperdown, NSW, Australia; ^5^The Charles Perkins Centre, University of Sydney, Camperdown, NSW, Australia; ^6^School of Life and Environmental Sciences, University of Sydney, Camperdown, NSW, Australia; ^7^Department of Mathematics and Statistics, Macquarie University, Sydney, NSW, Australia

**Keywords:** gut microbiota, gastrointestinal microbiome, longitudinal, progression, device-assisted therapies, levodopa-carbidopa intestinal gel, deep brain stimulation, Parkinson’s disease

## Abstract

**Background:**

Altered gut microbiome (GM) composition has been established in Parkinson’s disease (PD). However, few studies have longitudinally investigated the GM in PD, or the impact of device-assisted therapies.

**Objectives:**

To investigate the temporal stability of GM profiles from PD patients on standard therapies and those initiating device-assisted therapies (DAT) and define multivariate models of disease and progression.

**Methods:**

We evaluated validated clinical questionnaires and stool samples from 74 PD patients and 74 household controls (HCs) at 0, 6, and 12 months. Faster or slower disease progression was defined from levodopa equivalence dose and motor severity measures. 19 PD patients initiating Deep Brain Stimulation or Levodopa-Carbidopa Intestinal Gel were separately evaluated at 0, 6, and 12 months post-therapy initiation.

**Results:**

Persistent underrepresentation of short-chain fatty-acid-producing bacteria, *Butyricicoccus, Fusicatenibacter, Lachnospiraceae ND3007 group*, and *Erysipelotrichaceae UCG-003*, were apparent in PD patients relative to controls. A sustained effect of DAT initiation on GM associations with PD was not observed. PD progression analysis indicated that the genus *Barnesiella* was underrepresented in faster progressing PD patients at *t* = 0 and *t* = 12 months. Two-stage predictive modeling, integrating microbiota abundances and nutritional profiles, improved predictive capacity (change in Area Under the Curve from 0.58 to 0.64) when assessed at Amplicon Sequence Variant taxonomic resolution.

**Conclusion:**

We present longitudinal GM studies in PD patients, showing persistently altered GM profiles suggestive of a reduced butyrogenic production potential. DATs exerted variable GM influences across the short and longer-term. We found that specific GM profiles combined with dietary factors improved prediction of disease progression in PD patients.

## Introduction

Parkinson’s disease (PD) is a progressive multisystem disorder that contributes to significant morbidity and healthcare burden (GBD 2016 Parkinson’s Disease Collaborators, 2018), in addition to rendering a negative impact to patient and caregiver quality of life (QoL) (Schrag et al., 2000, 2001). With increased recognition of prodromal non-motor symptoms (NMS) in PD, particularly gastrointestinal dysfunction (Postuma et al., 2012; Durcan et al., 2019), an understanding of the importance of the gut-brain-axis as an integral bi-directional communication between the Enteric Nervous System (ENS) and the brain, which may facilitate the spread of α-synuclein (α-syn) pathology *via* a caudo-rostral gradient (Bullich et al., 2019; Lubomski et al., 2020c), has emerged. Altered abundance of numerous microbe taxa is well recognized between PD and household control (HC) gut microbiomes (GM). These differences have been associated with a variety of disease severities (Barichella et al., 2019), proposed to be mediated through an influence on gut permeability and colonic inflammation, which may facilitate α-syn aggregation and propagation in the local gastrointestinal environment (Bullich et al., 2019; Lubomski et al., 2020c; Romano et al., 2021). Furthermore, evidence suggests that certain microbiota, namely *Lactobacillus* and *Enterococcus faecalis*, can affect metabolism of Levodopa through increased tyrosine decarboxylase gene expression (Maini Rekdal et al., 2019; van Kessel et al., 2019, 2021) and could be exploited as a therapeutic target to improve Levodopa efficacy (Lubomski et al., 2019). Likewise, it is increasingly recognized that certain PD treatments, in particular the device-assisted therapies (DATs) can influence the GM *via* the gut-brain-axis, either through direct influence on the gut [Levodopa-Carbidopa Intestinal Gel (LCIG)] and or remote to the gut [Deep Brain Stimulation (DBS)] (Lubomski et al., 2020c, 2021c).

A meta-analysis of ten international cross-sectional PD GM datasets utilizing 16S rRNA-gene sequencing has recently shown overrepresentation of the genera *Lactobacillus, Akkermansia*, and *Bifidobacterium*, whilst underrepresentation of bacteria belonging to the *Lachnospiraceae* family and the *Faecalibacterium* genus (Romano et al., 2021). While all of these microbiota produce SCFAs, there are distinct differences in the health impacts of the metabolites produced (Aho et al., 2021; Baert et al., 2021). Lower levels of fecal SCFAs in PD patients have been hypothesized to be a consequence of a decreased abundance of SCFA-producing bacteria (Unger et al., 2016; Aho et al., 2021; Tan et al., 2021), which consequently causes increased gut permeability, inflammation (Keshavarzian et al., 2015) and gastrointestinal dysfunction in PD patients (Lubomski et al., 2020b).

There is a pressing need to develop reliable biomarkers for PD, empowering clinicians to diagnose disease early, distinguish PD from other parkinsonian syndromes, monitor treatment response and importantly, monitor PD progression (Emamzadeh and Surguchov, 2018). Accordingly, the GM has been proposed as a potentially suitable biomarker for PD (Romano et al., 2021). As gastrointestinal dysfunction and diet have major influences on the GM then these might also be integrated to create models for better clinical indication. While the literature supports altered GM compositions between PD patients and HCs, there is a paucity of studies assessing putative indicator taxa as chronic features of the PD microbiome. The aims of this study were threefold; firstly to characterize longitudinal GM changes in a cohort of PD patients and HCs over a year, secondly to evaluate whether characterization of GM profiles can predict PD progression, and thirdly to evaluate the GM composition for a year after DAT initiation.

## Materials and Methods

### Study Settings and Subjects

Subjects were recruited from the movement disorder and neurology clinics at Royal North Shore Hospital, Sydney, Australia, as reported in our previous studies (Lubomski et al., 2020a,b, 2021a,b,c; 2022). Inclusion criteria: being managed by a specialist neurologist, a clinical diagnosis of idiopathic PD according to the UK Parkinson’s Disease Society Brain Bank Diagnostic Criteria (Hughes et al., 1992), and >18 years of age. The HC inclusion criteria: exhibiting no clinical indication of PD, a spouse or close relative residing in the same household with similar dietary habits to their respective PD relative, and >18 years of age. Exclusion criteria included secondary Parkinsonism, medical or surgical disorders preventing completion of questionnaires, tube feeding, and significant cognitive impairment demonstrated by incapacity to provide consent. Ethical approval was granted by the Northern Sydney Local Health District Human Research Ethics Committee and the North Shore Private Hospital ethics committee (HREC/18/HAWKE/109, NSPHEC 2018-LNR-009 respectively).

### Data Collection and Longitudinal Evaluation

Parkinson’s disease and HC participants attending clinics between June 2018 – June 2019 were recruited to complete self-administered questionnaires, as well as providing stool and blood samples over a 1-year period, at 0, 6, and 12 months timepoints. Our previously described DAT cohort (Lubomski et al., 2021c), also provided a pre-DAT initiation stool sample taken 2 weeks prior to initiating DBS and LCIG (timepoint 0) in this study (Supplementary Figure 1), allowing definition of a pre-treatment GM. A 90% retention rate for DAT and existing therapy longitudinal PD participants was achieved, with 91% retention for HC participants, over the three time intervals. All participants had not received antibiotics or probiotic supplements for at least 1-month prior to each stool sample collection. The methodology explaining the DAT cohort in further detail has been reported previously (Lubomski et al., 2021c). Stool samples were assessed according to the Bristol Stool Scale (BSS) (Lewis and Heaton, 1997), whilst non-fasting blood samples were assessed for standard liver function tests, including albumin, in addition to non-fasting lipid profiles, Erythrocyte Sedimentation Rate and C-Reactive Protein (Table 1).

**TABLE 1:** Existing therapies cohort demographic and clinical characteristics.

	Timepoint	Parkinson’s disease (Existing Therapies)	Household control	Test statistic (df)	*p*-value
Number of patients (*n* = )	Baseline 6 Months 12 Months	82 76 74	81 75 74		
Age at Baseline, (years) [*SD*, Range]		67.2 [12.7, 35–88]	62.4 [15.6, 18–90]	*t* = 2.2 (161)^∧^	**0.031**
Gender, (%)					
Male	Baseline	57.3	32.1	χ^2^ = 10.5 (1)^∞^	**0.001**
Female	6 Months 12 Months	42.7 59.2 40.8 58.1 41.9	67.9 32.0 68.0 32.4 67.6	χ^2^ = 11.3 (1)^∞^ χ^2^ = 9.8 (1)^∞^	**0.001** **0.002**
Marital status (%)				χ^2^ = 5.4 (2)^∞^	0.143
Married/*De facto*		74.4	85.2		
Single		9.8	9.9		
Widowed/Separated		15.9	4.9		
Ethnicity (%)				χ^2^ = 1.8 (3)^∞^	0.619
Caucasian		78.0	79.0		
Asian		3.7	6.2		
Middle Eastern		6.1	2.5		
Other		12.2	12.3		
Body Mass Index, [*SD*]	Baseline 6 Months 12 Months	25.9 [4.9] 25.9 [5.1] 26.2 [5.1]	26.2 [4.6] 26.0 [4.2] 25.9 [4.2]	*t* = –0.3 (161)^∧^ *t* = –0.1 (149)^∧^ *t* = –0.3 (146)^∧^	0.752 0.968 0.734
Baseline Last Antibiotic Use (months), [*SD*, Range]		23.7 [37.9, 1–280]	25.7 [37.8, 1–288]	*t* = –0.4 (161)^∧^	0.728
Baseline smoking history (%)					
Current smoker		2.4	3.8	χ^2^ = 0.2 (1)^∞^	0.630
Prior smoker		40.2	33.8	χ^2^ = 0.7 (1)^∞^	0.392
Pack year history, [*SD*]		13.7 [14.3]	14.3 [14.6]	*t* = –0.1 (58)^∧^	0.868
Baseline alcohol consumption (%)		74.1	87.7	χ^2^ = 4.8 (1)^∞^	**0.028**
<Weekly		24.7	27.2	χ^2^ = 0.1 (1)^∞^	0.720
Several times weekly		32.1	33.3	χ^2^ = 0.3 (1)^∞^	0.867
Daily		19.8	28.4	χ^2^ = 1.7 (1)^∞^	0.198
Baseline caffeine consumption (Coffee/Tea), (%)		86.6	91.4	χ^2^ = 0.9 (1)^∞^	0.331
Number of daily cups, [*SD*]		2.4 [1.7]	3.1 [1.8]	*t* = –2.4 (161)^∧^	**0.016**
Baseline dietary intake					
Vegetarian diet (%)		1.2	2.5	χ^2^ = 0.4 (1)^∞^	0.546
Energy (kcal/day), [*SD*]		11152 [5431]	10188 [4799]	*t* = 1.2 (161)^∧^	0.233
Protein (g/day), [*SD*]		117.3 [73.1]	116.7 [74.5]	*t* = 0.1 (161)^∧^	0.965
Fat (g/day), [*SD*]		101.4 [48.9]	95.7 [43.6]	*t* = 0.8 (161)^∧^	0.437
Carbohydrate (g/day), [*SD*]		280.1 [154.0]	232.2 [124.9]	*t* = 2.2 (161)^∧^	**0.032**
Total sugars (g/day), [*SD*]		154.3 [88.9]	118.7 [60.6]	*t* = 2.9 (161)^∧^	**0.003**
Fiber (g/day), [*SD*]		40.3 [26.2]	38.1 [22.7]	*t* = 0.6 (161)^∧^	0.574
Baseline history of diabetes (%)		3.7	6.2	χ^2^ = 0.6 (1)^∞^	0.458
Gastrointestinal symptoms					
Cleveland constipation score, [*SD*]	Baseline 6 Months 12 Months	6.9 [4.5] 7.0 [4.9] 6.3 [4.5]	3.1 [2.9] 3.2 [2.5] 2.8 [2.5]	*t* = 6.4 (161)^∧^ *t* = 5.9 (149)^∧^ *t* = 5.8 (146)^∧^	**<0.001** **<0.001** **<0.001**
Constipation score as per ROME IV criteria, [*SD*]	Baseline 6 Months 12 Months	4.2 [3.5] 4.1 [3.5] 3.8 [2.9]	1.1 [1.4] 1.4 [2.0] 1.2 [1.5]	*t* = 7.3 (161)^∧^ *t* = 5.8 (149)^∧^ *t* = 6.5 (146)^∧^	**<0.001** **<0.001** **<0.001**
Functional constipation as per ROME IV criteria (%)	Baseline 6 Months 12 Months	75.6 78.9 71.6	28.4 33.3 36.5	χ^2^ = 36.4 (1)^∞^ χ^2^ = 31.9 (1)^∞^ χ^2^ = 18.4 (1)^∞^	**<0.001** **<0.001** **<0.001**
Bristol stool score, [*SD*]	Baseline 6 Months 12 Months	3.2 [1.6] 2.6 [1.2] 2.6 [1.2]	3.9 [1.3] 3.2 [1.4] 3.4 [1.3]	*t* = –2.7 (161)^∧^ *t* = –2.8 (149)^∧^ *t* = –4.0 (149)^∧^	**0.006** **0.006** **<0.001**
Leeds dyspepsia questionnaire (LDQ) score, [*SD*]	Baseline 6 Months 12 Months	8.3 [7.5] 6.8 [7.6] 6.7 [8.1]	4.6 [6.1] 3.2 [5.8] 3.5 [5.3]	*t* = 3.4 (161)^∧^ *t* = 3.3 (149)^∧^ *t* = 2.8 (146)^∧^	**0.001** **0.001** **0.006**
Chronic pain over last 3 months, (%)	Baseline 6 Months 12 Months	69.5 56.6 60.8	39.5 42.7 48.6	χ^2^ = 14.8 (1)^∞^ χ^2^ = 2.9 (1)^∞^ χ^2^ = 2.2 (1)^∞^	** < 0.001** 0.087 0.137
Pain score (Visual Analogue Scale) [*SD*]	Baseline 6 Months 12 Months	4.9 [2.5] 5.0 [1.9] 4.7 [2.3]	3.9 [1.7] 3.9 [2.3] 4.2 [2.1]	*t* = 1.9 (87)^∧^ *t* = 2.2 (74)^∧^ *t* = 1.0 (79)^∧^	0.052 **0.028** 0.306
International Physical Activity Questionnaire (IPAQ) Score (MET-minutes/week), [*SD*]	Baseline 6 Months 12 Months	2069.4 [1775.3] 1644.5 [1614.6] 1493.9 [1321.1]	2942.4 [2620.9] 2796.7 [2380.8] 2628.1 [2440.1]	*t* = –2.5 (161)^∧^ *t* = –3.5 (149)^∧^ *t* = –3.7 (146)^∧^	**0.014** **0.001** **<0.001**
Depression characteristics					
Beck’s depression inventory total score [*SD*]	Baseline 6 Months 12 Months	11.0 [7.9] 11.6 [8.4] 12.1 [9.0]	5.2 [5.5] 5.5 [5.9] 5.7 [5.7]	*t* = 5.4 (161)^∧^ *t* = 5.2 (149)^∧^ *t* = 5.1 (146)^∧^	**<0.001** **<0.001** **<0.001**
Clinically depressed (>13 for Parkinson’s disease and > 9 for control groups) (%)	Baseline 6 Months 12 Months	35.4 39.5 37.8	21.0 20.0 19.4	χ^2^ = 4.2 (1)^∞^ χ^2^ = 6.8 (1)^∞^ χ^2^ = 7.2 (1)^∞^	**0.041** **0.009** **0.001**
Montreal cognitive assessment (MoCA), [*SD*]					
MoCA total score, (/30)	Baseline 6 Months 12 Months	24.8 [4.5] 24.1 [5.2] 24.3 [4.9]	27.6 [2.6] 27.9 [2.8] 27.7 [2.6]	*t* = –4.9 (161)^∧^ *t* = –5.7 (149)^∧^ *t* = –5.3 (146)^∧^	**<0.001** **<0.001** **<0.001**
Mild cognitive impairment (< 26/30), (%)	Baseline 6 Months 12 Months	45.1 51.3 48.6	18.5 12.0 14.9	χ^2^ = 13.3 (1)^∞^ χ^2^ = 26.9 (1)^∞^ χ^2^ = 19.5 (1)^∞^	**<0.001** **<0.001** **<0.001**
Parkinson’s disease dementia (<21/30), (%)	Baseline 6 Months 12 Months	13.4 21.1 17.6	– – –		
36 - Item Short Form Health Survey (Quality of Life Assessment) [*SD*]					
Health Change Over Last Year	Baseline 6 Months 12 Months	42.4 [21.7] 42.1 [19.2] 41.6 [19.5]	50.6 [16.3] 52.4 [18.1] 51.0 [17.9]	*t* = –2.7 (146)^∧^ *t* = –3.4 (146)^∧^ *t* = –3.1 (146)^∧^	**0.007** **0.001** **0.003**
Physical Component Summary	Baseline 6 Months 12 Months	53.9 [23.0] 51.6 [22.9] 52.3 [22.5]	79.9 [17.7] 79.6 [16.4] 79.1 [17.4]	*t* = –8.1 (146)^∧^ *t* = –8.6 (146)^∧^ *t* = –8.1 (146)^∧^	**<0.001** **<0.001** **<0.001**
Mental component summary	Baseline 6 Months 12 Months	63.6 [20.7] 60.7 [22.3] 61.8 [23.2]	80.8 [17.4] 80.5 [14.8] 79.6 [17.1]	*t* = –5.7 (146)^∧^ *t* = –6.1 (146)^∧^ *t* = –5.3 (146)^∧^	**<0.001** **<0.001** **<0.001**
Baseline biochemical characteristics, [*SD*]					
Erythrocyte sedimentation rate		10.0 [14.8]	9.5 [10.4]	*t* = –0.2 (161)^∧^	0.821
C-reactive protein		3.6 [10.8]	2.2 [2.4]	*t* = 1.1 (161)^∧^	0.280
Total cholesterol		4.8 [1.0]	5.2 [1.1]	*t* = –2.0 (161)^∧^	**0.043**
Low density lipoprotein		2.7 [0.7]	2.9 [0.9]	*t* = –1.4 (161)^∧^	0.164
High density lipoprotein		1.5 [0.4]	1.6 [0.4]	*t* = –1.5 (161)^∧^	0.130
Triglycerides		1.3 [1.1]	1.5 [0.9]	*t* = –1.0 (161)^∧^	0.316
Random glucose		5.8 [0.5]	5.9 [0.9]	*t* = –0.7 (161)^∧^	0.502
HbA1c%		5.3 [0.3]	5.4 [0.6]	*t* = –0.9 (161)^∧^	0.343
Albumin		39.5 [2.9]	39.8 [3.1]	*t* = –0.7 (161)^∧^	0.484
Baseline Parkinson’s disease characteristics					
Age at diagnosis, (years) [*SD*]		58.8 [14.7]			
Parkinson’s disease duration, (years) [*SD*]		8.6 [6.8]			
Baseline Parkinson’s disease phenotype, (%)					
Tremor dominant		29.3			
Postural instability and gait impairment		19.5			
Akinetic rigid		39.0			
Young onset (<40 years)		12.2			
Late onset (>60 years)		53.7			
Baseline disease Complications, (%)					
Motor fluctuations		47.6			
Dyskinesia		52.4			
Wearing off		76.8			
Impulse control disorder		18.3			
Baseline non-motor symptoms, (%)					
Hyposmia		74.4			
REM sleep behavior disorder		47.6			
Levodopa equivalent daily dose (mg), [*SD*]	Baseline 6 Months 12 Months	716.8 [505.4] 745.1 [475.8] 796.7 [563.7]			
MDS unified Parkinson’s disease rating scale – III, (“on” state) [*SD*]	Baseline 6 Months 12 Months	30.2 [16.1] 34.5 [12.4] 36.2 [14.6]			
Parkinson’s disease therapy, (%)					
Medication Naïve Oral Levodopa	Baseline 6 Months 12 Months Baseline 6 Months 12 Months	(*n* = 5) 6.1 (*n* = 3) 3.9 (*n* = 2) 2.7 (*n* = 71) 86.6 (*n* = 67) 88.2 (*n* = 66) 89.2			
Dopamine agonist	Baseline 6 Months 12 Months	(*n* = 23) 28.0 (*n* = 19) 25.0 (*n* = 19) 25.7			
Monoamine oxidase B inhibitor	Baseline 6 Months 12 Months	(*n* = 15) 18.3 (*n* = 13) 17.1 (*n* = 14) 18.9			
Anticholinergic	Baseline 6 Months 12 Months	(*n* = 12) 14.6 (*n* = 10) 13.2 (*n* = 9) 12.2			
Catechol-*O*-methyl transferase inhibitor	Baseline 6 Months 12 Months	(*n* = 15) 18.3 (*n* = 13) 17.1 (*n* = 12) 16.2			
Amantadine	Baseline 6 Months 12 Months	(*n* = 10) 12.2 (*n* = 8) 10.5 (*n* = 7) 9.5			
Apomorphine (Subcutaneous infusion) Levodopa-carbidopa intestinal gel Deep brain stimulation	Baseline 6 Months 12 Months Baseline 6 Months 12 Months Baseline 6 Months 12 Months	(*n* = 2) 2.4 (*n* = 1) 1.3 (*n* = 1) 1.4 (*n* = 9) 11.0 (*n* = 7) 9.2 (*n* = 7) 9.5 (*n* = 11) 13.4 (*n* = 9) 11.8 (*n* = 9) 12.2			
Quality of life					
PDQ-39 summary index [*SD*]	Baseline 6 Months 12 Months	26.9 [16.8] 26.2 [16.4] 28.2 [18.2]			
MDS non-motor symptoms score (NMSS) – total score, [*SD*]	Baseline 6 Months 12 Months	56.1 [35.8] 68.3 [42.7] 67.0 [43.1]			

*^Independent sample t-test. ^∞^Pearson’s chi-squared test. df, degrees of freedom; SD, standard deviation.*

*Bold values indicate p values with statistical significance, p < 0.05.*

Information regarding socio-demographic factors, lifestyle, clinical management and comorbidities was collected from all participants using validated surveys at all three intervals, in addition to a validated comprehensive Food Frequency Questionnaire (Barclay et al., 2008; Palavra et al., 2021). The dietary questionnaire allowed extrapolation of nutritional intake, including energy, protein, fat, carbohydrate, sugar and fiber intake. Patients completed the Leeds Dyspepsia Questionnaire (LDQ) (Moayyedi et al., 1998), assessing upper gastrointestinal symptoms. Constipation severity and gut motility were evaluated by the Rome-IV criteria (Sood and Ford, 2016) and the Cleveland Constipation Score (CCS) (Agachan et al., 1996). QoL was assessed by the Parkinson’s Disease Questionnaire-39 (PDQ-39) (Jenkinson et al., 1997) and the Short Form Health Survey (SF-36) (Ware and Sherbourne, 1992), whilst chronic pain severity was assessed by the Visual Analog Scale (McCormack et al., 1988). Non-motor symptoms were assessed by the Movement Disorder Society-Non-Motor Symptom Score (MDS–NMSS) (Chaudhuri et al., 2007), physical activity was estimated by the International Physical Activity Questionnaire (IPAQ) (Hagstromer et al., 2006), mood by the Beck Depression Inventory (BDI) (Beck et al., 1961) and cognitive function by the Montreal Cognitive Assessment (MoCA) (Nasreddine et al., 2005). Clinical motor assessments were performed by one neurologist during a patient’s “on” state, as an objective measure of the prevailing motor function, in accordance with the Movement Disorder Society – Unified Parkinson’s Disease Rating Scale – Part III (MDS-UPDRS III) criteria (Goetz et al., 2008). PD phenotype (tremor dominant or akinetic rigid) was determined using MDS guidelines for assigning such phenotypes (Stebbins et al., 2013). Medications were compared using standard calculations of daily levodopa equivalent dose (LED) (Tomlinson et al., 2010).

Patients on existing therapeutic regimes were subcategorized into faster and slower progressors, defined by the net differences in MDS-UPDRS-III and LED scores, being objective clinician-derived scores of relative disease progression over the 12-month period. Individuals who presented with an increase in both MDS-UPDRS-III and LED over the 12 months interval were classed as faster progressors, whilst for those whose MDS-UPDRS-III and/or LED remained the same or slightly decreased over the 12 months interval, they were classed as slower progressors.

### Stool DNA Extraction and 16S Ribosomal RNA Amplicon Sequencing

Stool samples were snap frozen upon receipt, with total fecal DNA isolated within 2 months of receiving stool samples. DNA was extracted using an optimized protocol for the MP Biomedicals FastDNA™ SPIN Kit for Feces (MP Biomedicals, Santa Ana, CA, United States). DNA integrity was confirmed by polymerase chain reaction using universal primers to the V3–V4 regions (341f and 805r) and whole genome (27f and 1492r) of bacterial 16S ribosomal DNA (Weisburg et al., 1991; Klindworth et al., 2013).

16S rRNA V3-V4 amplicon sequencing was performed by the Ramaciotti Centre for Genomics (University of New South Wales, Sydney, Australia). Sequencing libraries were generated using standard V3–V4 primers (341f and 805r; Weisburg et al., 1991) and a two-stage amplicon and indexing PCR with KAPA HiFi polymerase to generate 300 bp paired-end reads. Libraries were cleaned up after each PCR using Ampure XP beads and normalized using the Applied Biosciences SequalPrep™ Plate Normalization kit (Thermo Fisher, Waltham, MA, United States). Sequencing was performed on an Illumina MiSeq platform using MiSeq v3 chemistry with PhiX control v3. Internal sequencing controls included replicate patient stool DNA samples and the ZymoBIOMICS Microbial Community DNA Standard (Zymo Research, Irvine, CA, United States) for validation of sequencing batches.

### Computational and Statistical Analyses

#### Statistical Data Analysis

Clinical data comparisons between groups were performed by Student’s *t*-tests and χ^2^ tests for quantitative and categorical variables, respectively, using SPSS, version 26 (SPSS Inc, Chicago, IL, United States). Variables were assessed by Levene’s test to ensure homogeneity of variances. *p* < 0.05 was considered statistically significant. All statistical comparisons and data visualizations were performed with R (v.3.5.1) and figures were generated with ggplot2 (v.3.1.0).

#### Data Pre-processing

The R-package dada2 (v.1.14.1) was used to process sequence data into amplicon sequences variant (ASV) tables. The forward and reverse error profiles were trimmed to maintain high quality (Supplementary Figure 2). The sequences were trimmed from 37 to 270 bp and 10 to 222 bp in forward and reverse reads, respectively. Subsequently, the sequence data was de-replicated to remove redundancy and combine all identical sequence reads into a “unique sequence.” The dada2 method removed all substitution and indel errors. The resulting sequence was further merged by removing paired sequences without perfect overlap. Finally, the chimeras were removed by comparing the inferred sequence to others and removing those that could be reproduced by stitching together two more abundant sequences. ASVs were assigned to taxonomic groups according to the Silva (v.138) reference database, which may slightly vary the taxonomic classification compared to earlier studies, due to greater resolution in taxa assignment from this version of the updated database. The ASV tables were further filtered with a prevalence of more than 10%, i.e., an ASV present in at least 5% of the total sample or equivalently present in less than 26 samples, to avoid noise or present rare taxa among the samples.

#### Microbiome Community Analysis

Alpha diversity metrics including the Shannon index and taxon richness were calculated for each sample and a linear mixed effect (lme) model was used to determine changes associated with time and PD treatment. Beta-diversity was analyzed using the R-package vegan (v.2.5–3) to assess turnover between samples using a range of metrics, i.e., Bray–Curtis (BC) dissimilarity, unweighted and weighted unifrac distance. A Principal Coordinate Analysis (PCoA) was used for both dimension reduction and visualizing the relationships among samples. To assess the significance of beta diversity between treatments or time intervals, a permutational multivariate analysis of variance (PERMANOVA) model was used. This model is fitted using the adonis2 function in the vegan package with the argument “by” = “margins” and “perm” = 9999 for all comparisons. To compare the compositional difference among all time intervals, an ANOVA-like differential expression (ALDE) model was used at four levels of taxonomic resolution (phylum, order, family and genus). The data were compared between different sampling time points (*t* = –2 vs. *t* = 0, *t* = 0 vs. *t* = 6, *t* = 6 vs. *t* = 12, and *t* = 0 vs. *t* = 12) for the two DAT groups, with *t* = 0 defined as baseline. The model was fit using the R package ALDEx2 (v1.16). In the fitting of the ALDE model, parameter “mc.samples” were set to 128 and “denom” = All. For the comparison of difference between PD and HC groups, a similar ALDE model was fit at *t* = 0. Comparison of relative abundance of different bacterial taxa between the existing therapies PD and HC groups at *t* = 0, 6, and 12 were represented by an ALDEx2 model, at the same four taxonomic levels and utilized the Benjamini–Hochberg procedure to correct the *p*-value for multiple testing. Existing PD therapy patients were categorized into faster and slower progressors, informed by their differential *t* = 0 and 12 months LED and MDS-UPDRS-III scores. PICRUSt2 analysis assessed the predictive functional potential of a bacterial community on the basis of marker gene sequencing profiles (Douglas et al., 2020).

#### Prediction Analysis

A combined central log transformation with support vector machine model to predict faster progressing PD patients using microbiota and clinical covariates at different taxonomic levels was built. Model performance at each taxonomic level was based on applying the concept of leave-one-out cross validation to calculate the area under the receiver operating characteristics curve (LOOCV-AUC) (Lubomski et al., 2022).

A two-stage classification model was constructed with both the macronutrient intake data and microbiota profiles. The first stage partitioned the samples into two groups based on a given variable representing the macronutrient intake. This cutoff value was used as the splitting point corresponding to the decision tree, with macronutrient intake as the only node. The split was based on the maximum information gain from the entire cohort versus splitting to two sub-cohorts. The second stage built different random forest models to predict PD progression for each of the two sub-cohorts. This two-stage model was repeated for different macronutrients as partitioning nodes and calculated the corresponding LOOCV-AUC for each two-stage model.

## Results

### Demographic Characteristics of the Longitudinal and Device-Assisted Therapies Cohorts

In this paper we report on two distinct patient cohorts: (1) patients receiving existing therapy regimes, sampled at three timepoints (0, 6, 12 months) consisting of *n* = 148 participants; 74 PD patients and 74 HCs at 12-month completion (Table 1 and Supplementary Figure 1). (2) 19 initiating DAT patients [9 (DBS) and 10 (LCIG)] who had been previously described (Lubomski et al., 2021c), were sampled at three timepoints (0, 6, 12 months) to assess for delayed GM influences after DAT initiation (Table 2 and Supplementary Figure 1). 58.1% of the existing therapy PD participants were male with a mean age of 67.2 years [range 35–88, standard deviation (*SD*) 12.7], whilst 32.4% of the HC’s were male, with a mean age of 62.4 years [range 18–90, (*SD* 15.6)], Table 1. Of the combined longitudinal cohort (PD and HC), 80% of the participants were married and identified themselves of Caucasian ancestry. Further demographic characteristics of this cohort are presented in Table 1.

**TABLE 2:** Device – assisted therapies Parkinson’s disease patient clinical characteristics.

	Timepoint	DBS cohort	LCIG cohort	Test statistic (df)	*p*-value
Number of patients (*n* = )*	Baseline 6 Months 12 Months	10 9 9	11 10 10		
Age at baseline, (years) [*SD*, Range]*		65.8 [9.2]	67.0 [11.0]	*t* = 0.8 (19)^∧^	0.790
Age at diagnosis, (years) [*SD*]*		53.6 [7.2]	56.3 [9.1]	*t* = 0.7 (19)^∧^	0.466
Parkinson’s disease duration, (years) [*SD*]*		12.2 [4.2]	10.6 [4.9]	*t* = –0.8 (19)^∧^	0.423
Parkinson’s disease phenotype (%)*				χ^2^ = 2.9 (3)^∞^	0.400
Tremor dominant		50.0	18.2		
Postural instability and gait impairment		20.0	27.3		
Akinetic rigid		30.0	45.5		
Young onset (<40 years)		0	9.1		
Late onset (>60 years)		30.0	36.4	χ^2^ = 0.1 (3)^∞^	0.757
Disease complications at baseline (%)*					
Motor fluctuations		100.0	100.0		
Dyskinesia		70.0	90.9	χ^2^ = 1.5 (1)^∞^	0.223
Wearing off		100.0	100.0		
Impulse control disorder		20.0	27.3	χ^2^ = 1.5 (1)^∞^	0.696
Non-motor symptoms at baseline (%)*					
Hyposmia		70.0	72.7	χ^2^ = 0.1 (1)^∞^	0.890
REM sleep behavior disorder		40.0	63.6	χ^2^ = 1.2 (1)^∞^	0.279
Levodopa equivalent daily dose (mg) [*SD*]*	Baseline 6 Months 12 Months	1175.7 [213.9] 620.8 [284] 618.1 [208.3]	1404.4 [372.0] 1569.9 [556.6] 1623.3 [637.1]	*t* = 1.7 (19)^∧^ *t* = 4.6 (17)^∧^ *t* = 4.5 (17)^∧^	0.105 **<0.001** **<0.001**
MDS unified Parkinson’s disease rating scale – III, (“on” state) [*SD*]*	Baseline 6 Months 12 Months	35.5 [13.6] 26.1 [13.1] 27.7 [12.9]	51.2 [22.2] 44.0 [23.6] 52.4 [19.1]	*t* = 1.9 (19)^∧^ *t* = 2.0 (17)^∧^ *t* = 3.3 (17)^∧^	0.069 0.060 **0.004**
Parkinson’s disease therapy, (%)*					
Oral levodopa	Baseline 6 Months 12 Months	100.0 100.0 100.0	100.0 60.0 90.0	χ^2^ = 4.6 (1)^∞^ χ^2^ = 9.5 (1)^∞^	**0.033** 0.330
Dopamine agonist	Baseline 6 Months 12 Months	60.0 55.6 55.6	63.6 60.0 60.0	χ^2^ = 0.3 (1)^∞^ χ^2^ = 0.1 (1)^∞^ χ^2^ = 0.1 (1)^∞^	0.864 0.845 0.845
Monoamine oxidase B inhibitor	Baseline 6 Months 12 Months	20.0 11.1 22.2	18.2 20.0 10.0	χ^2^ = 0.1 (1)^∞^ χ^2^ = 0.3 (1)^∞^ χ^2^ = 0.5 (1)^∞^	0.916 0.596 0.466
Anticholinergic	Baseline 6 Months 12 Months	0 0 0	9.9 10.0 10.0	χ^2^ = 0.9 (1)^∞^ χ^2^ = 0.9 (1)^∞^ χ^2^ = 0.9 (1)^∞^	0.329 0.330 0.330
Catechol-*O*-methyl transferase inhibitor	Baseline 6 Months 12 Months	40.0 0 0	45.5 10.0 10.0	χ^2^ = 0.1 (1)^∞^ χ^2^ = 0.9 (1)^∞^ χ^2^ = 0.9 (1)^∞^	0.801 0.330 0.330
Amantadine	Baseline 6 Months 12 Months	10.0 0 0	18.2 0 10.0	χ^2^ = 0.3 (1)^∞^ χ^2^ = 0.9 (1)^∞^	0.593 0.330
Apomorphine (subcutaneous infusion)	Baseline 6 Months 12 Months	0 0 0	36.4 0 0	χ^2^ = 4.5 (1)^∞^	**0.034**
Quality of life					
PDQ-39 summary index, [*SD*]*	Baseline 6 Months 12 Months	29.6 [11.9] 22.1 [12.9] 25.6 [14.1]	46.1 [17.0] 36.2 [15.9] 38.6 [19.9]	*t* = 2.6 (19)^∧^ *t* = 2.1 (17)^∧^ *t* = 1.7 (17)^∧^	**0.019** 0.050 0.124
MDS non-motor symptoms score (NMSS) – total score [*SD*]*	Baseline 6 Months 12 Months	69.4 [61.7] 62.2 [36.3] 51.8 [36.8]	106.4 [50.2] 80.8 [43.5] 86.3 [61.2]	*t* = 1.5 (19)^∧^ *t* = 1.0 (17)^∧^ *t* = 1.4 (17)^∧^	0.147 0.330 0.160
Gastrointestinal symptoms*					
Cleveland constipation score [*SD*]	Baseline 6 Months 12 Months	5.6 [2.8] 5.4 [3.1] 5.1 [2.4]	10.5 [5.7] 8.4 [5.0] 6.1 [5.7]	*t* = 2.4 (19)^∧^ *t* = 1.5 (17)^∧^ *t* = 0.5 (17)^∧^	**0.025** 0.147 0.635
Constipation score as per ROME IV criteria, [*SD*]	Baseline 6 Months 12 Months	4.3 [2.8] 2.9 [2.7] 4.0 [2.0]	5.9 [3.9] 4.1 [2.7] 3.9 [2.9]	*t* = 1.1 (19)^∧^ *t* = 0.9 (17)^∧^ *t* = –0.1 (17)^∧^	0.300 0.346 0.932
Functional constipation as per ROME IV criteria, (%)	Baseline 6 Months 12 Months	90.0 55.6 88.9	90.9 90.0 80.0	χ^2^ = 0.1 (1)^∞^ χ^2^ = 2.9 (1)^∞^ χ^2^ = 0.3 (1)^∞^	0.943 0.089 0.596
Bristol stool score [*SD*]	Baseline 6 Months 12 Months	1.9 [0.6] 2.3 [1.0] 1.8 [1.2]	2.3 [1.5] 2.5 [1.1] 3.4 [1.3]	*t* = 0.7 (19)^∧^ *t* = 0.4 (17)^∧^ *t* = 2.8 (17)^∧^	0.467 0.732 **0.011**
Leeds dyspepsia questionnaire (LDQ) score, [*SD*]	Baseline 6 Months 12 Months	7.2 [5.5] 6.3 [4.7] 8.4 [6.4]	9.6 [11.0] 5.6 [8.9] 5.7 [9.1]	*t* = 0.6 (19)^∧^ *t* = –0.2 (17)^∧^ *t* = –0.7 (17)^∧^	0.551 0.829 0.463
International physical activity questionnaire (IPAQ) score (MET-minutes/week) [*SD*]*	Baseline 6 Months 12 Months	1284.7 [887.0] 2073.5 [2109.8] 2232.3 [2243.1]	481.2 [501.7] 314.0 [224.5] 505.6 [515.2]	*t* = –2.6 (19)^∧^ *t* = –2.6 (17)^∧^ *t* = –2.3 (17)^∧^	**0.018** **0.018** **0.030**
Depression characteristics*					
Beck’s depression inventory total score, [*SD*]	Baseline 6 Months 12 Months	11.5 [8.8] 5.9 [4.5] 7.1 [4.2]	18.9 [12.5] 12.2 [11.5] 13.2 [16.6]	*t* = 1.5 (19)^∧^ *t* = 1.5 (17)^∧^ *t* = 1.1 (17)^∧^	0.136 0.141 0.301
Clinically depressed (>13 for Parkinson’s disease (%)	Baseline 6 Months 12 Months	30.0 22.2 0	72.7 40.0 20.0	χ^2^ = 3.8 (1)^∞^ χ^2^ = 0.7 (1)^∞^ χ^2^ = 2.0 (1)^∞^	0.050 0.405 0.156
Montreal cognitive assessment (MoCA), [*SD*]*					
MoCA total score, (/30)	Baseline 6 Months 12 Months	25.2 [2.5] 24.7 [6.5] 26.2 [1.6]	21.2 [7.4] 20.3 [6.3] 20.1 [7.3]	*t* = –1.6 (19)^∧^ *t* = –1.8 (17)^∧^ *t* = –2.4 (17)^∧^	0.117 0.084 **0.025**
Mild cognitive impairment (<26/30), (%)	Baseline 6 Months 12 Months	60.0 44.4 22.2	63.6 70.0 70.0	χ^2^ = 0.1 (1)^∞^ χ^2^ = 1.3 (1)^∞^ χ^2^ = 4.3 (1)^∞^	0.864 0.260 **0.037**
Parkinson’s disease dementia (<21/30), (%)	Baseline 6 Months 12 Months	0 11.1 0	45.5 60.0 40.0	χ^2^ = 5.9 (1)^∞^ χ^2^ = 4.9 (1)^∞^ χ^2^ = 4.6 (1)^∞^	**0.015** **0.027** **0.033**

^Independent sample t-test. ^∞^Pearson’s chi-squared test. df, degrees of freedom; SD, standard deviation. *This data is partially reproduced (Lubomski et al., 2021c).

*Bold values indicate p values with statistical significance, p < 0.05.*

Disease progression status was analyzed within the existing therapies cohort and was characterized by the difference in the combined MDS-UPDRS-III and LED scores over the 12-month period, identifying two groups, *n* = 39 faster and *n* = 40 slower progressing PD patients. Faster progressing PD patients had a younger mean age of 65.5 years (*SD* 10.4), whilst slower progressing patients had an older mean age of 71.1 years (*SD* 11.6), (*p* = 0.034) with no differences in sex between the groups (Table 3). No difference in the disease duration was noted between the groups, nor any difference in patient mean Body Mass Index (BMI), PDQ-39 Summary Index or constipation prevalence according to the Rome-IV criteria. Faster progressing patients had a lower mean MDS-UPDRS-III at baseline, 25.2 (*SD* 12.1) vs. 34.4 (*SD* 17.1) (*p* = 0.001). Although they showed a significantly greater increase in their motor severity scores over the 12-month interval; 12.8 (*SD* 9.0) vs. 1.4 (*SD* 15.4), (*p* = 0.001). In addition, they required a greater mean LED increase of 191.1mg (*SD* 236.8) compared to 40.7 mg (*SD* 149.3) (*p* = 0.001) (Table 3).

**TABLE 3:** Clinical variables of faster and slower progressing Parkinson’s patients receiving existing therapies.

	Timepoint	Faster progressor	Slower progressor	Test statistic (df)	*p*-value
Number of patients (*n* = )		34	40		
Age at baseline, (years) [*SD*, Range]		65.5 [10.4]	71.1 [11.6]	*t* = –2.2 (72)^∧^	**0.034**
Age at diagnosis, (years) [*SD*]		57.4 [13.5]	63.1 [13.6]	*t* = –1.8 (72)^∧^	0.078
Parkinson’s disease duration at baseline, (years) [*SD*]		8.3 [6.9]	8.2 [6.5]	*t* = 0.1 (72)^∧^	0.965
Gender, (%)					
Male		52.9	62.5	χ^2^ = 0.7 (1)^∞^	0.481
Female		47.1	37.5		
Levodopa equivalent daily dose (mg), [*SD*]	Baseline 6 Months 12 Months Δ 12 months	711.6 [532.2] 790.5 [505.3] 902.7 [628.3] 191.1 [236.8]	665.8 [480.5] 701.5 [461.2] 706.5 [492.6] 40.7 [149.3]	*t* = 0.4 (72)^∧^ *t* = 0.8 (72)^∧^ *t* = 1.5 (72)^∧^ *t* = 2.3 (72)^∧^	0.698 0.431 0.137 **0.001**
MDS unified Parkinson’s disease rating scale – III (“on” state) [*SD*]	Baseline 6 Months 12 Months Δ 12 months	25.2 [12.1] 34.9 [13.5] 38.1 [14.7] 12.8 [9.0]	34.4 [17.1] 35.4 [11.7] 35.8 [14.5] 1.4 [15.4]	*t* = -2.6 (72)^∧^ *t* = 0.2 (72)^∧^ *t* = 0.9 (72)^∧^ *t* = 4.1 (72)^∧^	**0.011** 0.870 0.347 **0.001**
Body mass index, [*SD*]	Baseline 6 Months 12 Months	25.8 [4.8] 25.9 [5.1] 25.9 [5.2]	26.2 [5.2] 26.2 [5.3] 26.4 [5.1]	*t* = -0.3 (72)^∧^ *t* = -0.3 (72)^∧^ *t* = –0.4 (72)^∧^	0.755 0.796 0.709
Functional constipation as per ROME IV criteria, (%)	Baseline 6 Months 12 Months	70.5 79.4 76.5	77.5 77.5 72.5	χ^2^ = 0.5 (1)^∞^ χ^2^ = 0.8 (1)^∞^ χ^2^ = 0.4 (1)^∞^	0.596 0.988 0.446
PDQ-39 summary index, [*SD*]	Baseline 6 Months 12 Months	26.8 [16.3] 28.4 [16.7] 29.9 [18.8]	26.7 [18.1] 24.2 [16.4] 26.8 [17.8]	*t* = 0.1 (72)^∧^ *t* = 1.1 (72)^∧^ *t* = 0.8 (72)^∧^	0.983 0.292 0.441

*^Independent sample t-test. ^∞^Pearson’s chi-squared test. df, degrees of freedom; SD, standard deviation.*

*Bold values indicate p values with statistical significance, p < 0.05.*

### Clinical Characteristics of the Existing Therapies Longitudinal Parkinson’s Disease and Household Control Cohort

Clinical characteristic relevant to PD or known to be associated with microbiome dynamics were explored and are presented in Table 1. No significant differences between participant BMI or diabetic comorbidity were observed between the PD and HC groups. The mean last antibiotic use in the combined cohort was 24.7 months (*SD* 76), with no difference between the groups; PD range = 1–280 months and HC range = 1–288 months. Patients with PD experienced increased constipation according to the Rome IV criteria, 71.6% vs. 36.5% (*p* < 0.001), as well as increased constipation severity [Rome IV constipation score 3.8 (*SD* 2.9) vs. 1.2 (*SD* 1.5), *p* = 0.001; CCS, 6.3 (*SD* 4.5) vs. 2.8 (*SD* 2.5), *p* < 0.001] (Table 1). PD patients also had significantly harder stool according to the BSS score [2.6 (*SD* 1.2) vs. 3.4 (*SD* 1.3), *p* < 0.001], likely reflecting slowed colonic transit times. PD patients also reported increased upper gastrointestinal symptoms, as assessed by the LDQ [6.7 (*SD* 8.1) vs. 3.5 (*SD* 5.3), *p* = 0.006] (Table 1). PD patients reported reduced physical activity, more prevalent and increased depression severity, higher proportions of cognitive impairment and lower QoL compared to HC participants (Table 1). Blood biochemistry results were within the expected healthy range in both groups. Mean macronutrient intake did not differ greatly between the PD and HC groups, aside from PD patients reporting increased carbohydrate intake [280.1 g/day (*SD* 154.0) vs. 232.2/day (*SD* 124.9), *p* = 0.032] and total sugar intake [154.3 g/day (*SD* 88.9) vs. 118.7g/day (*SD* 60.6), *p* = 0.001] (Table 1). Within the PD cohort, the mean age at diagnosis was 58.8 years (*SD* 14.7), with a mean disease duration of 8.6 years (*SD* 6.8) (Table 1). Approximately one third of all PD patients had either a tremor dominant or akinetic rigid phenotype. 47.6% patients reported motor fluctuations, 76.8% reported medications “wearing off” prior to their next dose and 52.4% also reported dyskinesias. Of the NMS, 74.4% reported hyposmia, 47.6% Rapid Eye Movement Sleep Behavior Disorder and 18.3% had an impulse control disorder. The mean LED was 796.7 mg (*SD* 563.7), whilst the mean ‘on’ MDS UPDRS-III score was 36.2 (*SD* 14.6). The mean MDS–NMSS was 67.0 (*SD* 43.1), whilst the PDQ-39 Summary Index was 28.2 (*SD* 18.2) (Table 1). 2.7% (n = 2) of PD patients remained medication naïve throughout the 12-month follow-up, whilst 89.2% utilized oral levodopa, 25.7% a dopamine agonist, 18.9% a monoamine oxidase inhibitor, 12.2% an anticholinergic, 16.2% a catechol-*O*-methyl transferase inhibitor, 9.5% amantadine, whereas utilization of existing DAT included 1.4% apomorphine subcutaneous infusions, 9.5% LCIG and 12.2% DBS. The utilization of all the existing therapies across the three-time intervals, as well as other PD patient demographic and clinical characteristics, are provided in Table 1.

### Microbiome Data

The total number of sequencing reads from the two cohorts was 25,856,340, with a mean total of 58,235 reads per sample (Supplementary Figure 2). These reads were assigned to 9474 ASVs. After filtering low abundance-ubiquity (<5% of all samples or appeared in less than 28 samples), the final dataset had 573 ASVs. These post-filtering ASVs were assigned to a reference taxonomy and found to represent 9 phyla, 15 orders, 31 families and 93 genera. The number of ASVs (presented in square brackets) that were assigned to each of these genera included*: Butyricicoccus* [10], *Fusicatenibacter* [2], *Lachnospiraceae ND3007 group* [2], *Erysipelotrichaceae UCG-003* [3]. Most of the variation between the cohorts was observed in relative abundance distributions at the genus and family levels.

### Alterations in the Gut Microbiota Between Parkinson’s Disease and Household Control Cohorts

#### Relative Abundance

**FIGURE 1:**
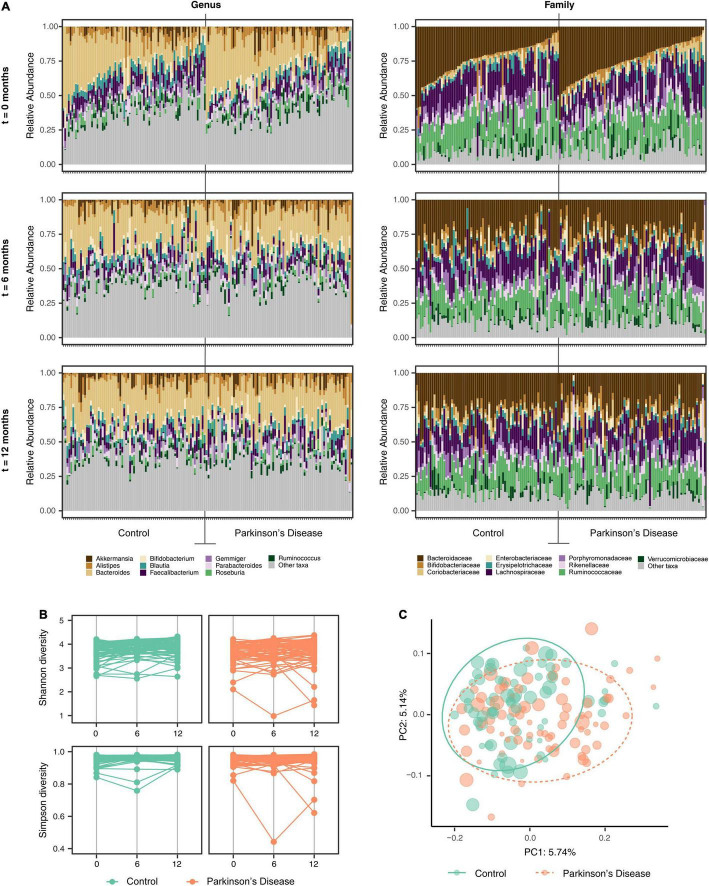
Alterations in the gut microbiota between the Parkinson’s disease (PD) and household control (HC) cohorts. **(A)** Individual participant mean relative abundance differences for the PD (*n* = 74) and HC (*n* = 74) groups at genus and family taxonomic levels at 0, 6, and 12 months intervals, were statistically different between the groups (ANOVA *p* < 0.01 genus, *p* < 0.01 family). **(B)** Alpha-diversity (Shannon and Simpson diversity) at the Amplicon Specific Variant (ASV) level for PD (*n* = 74) and HC (*n* = 74) participants across 0, 6, and 12 months intervals, showed significant alpha-diversity differences between the groups (ANOVA, *p* < 0.01 and *p* < 0.01 for Shannon and Simpson diversity, respectively). **(C)** Beta-diversity (bacterial richness between samples) explored by Principal Component Analysis (PCoA) with Bray–Curtis ordination at the ASV level was averaged for each participant and presented as a sphere relative to the variability of the diversity measure across the three time intervals. A statistically significant difference in beta diversity was noted between the two groups (PERMANOVA *p* < 0.01).

Comparison of the mean relative abundance at the genus and family levels for the 74 individual PD patients and 74 HC at baseline, 6 and 12 months are presented in Figure 1A and Supplementary Figure 3. Statistically significant relative abundance differences were noted between the PD and HC groups at genus, family, order and phylum taxonomic levels across the three-time intervals (PERMANOVA; *p* < 0.01 genus, *p* < 0.01 family, *p* < 0.01 order, *p* = 0.03 phylum).

#### Diversity

To examine if differences in the complexity of the community samples existed over time (disease progression changes) or between cohorts (disease state as a driver of community), we explored a range of diversity metrics. Alpha diversity represents partitioning of biological space in each community (sample). Assessment of species richness indicated a Chao1 at ASV level (*p* = 0.033). A significant difference in the mean alpha diversity between PD and HC cohorts at baseline, 6 and 12 months evaluated at the ASV, genus and family taxonomic levels was observed, (ANOVA, *p* < 0.01 and *p* < 0.01 for Shannon and Simpson diversity respectively) (Figure 1B).

Alpha diversity metrics do not specify differences in taxa within a community. To test for this, we explored beta diversity (diversity differences between samples) *via* Principal Component Analysis (PCoA) with multiple metrics (Bray–Curtis dissimilarity, unweighted Unifrac and weighted Unifrac). PCoA ordinations of Bray–Curtis distances at the ASV level showed a small, but statistically significant, difference in beta diversity between the PD and HC groups (*n* = 148) at baseline, 6 and 12 months (Supplementary Figure 4).

Each participant’s beta diversity was averaged over all time points and presented as pooled samples (PERMANOVA, *p* < 0.01) (Figure 1C). Evaluating cohabitant effect, when matching available PD and HC cases at all of the time intervals, for potential confounding from geographic or household factors, variations in beta diversity between the two cohorts were identified that were suggestive of a disease-related GM effect rather than a geographic or household effect (PERMANOVA, *p* < 0.01) (Supplementary Figure 5). Bray–Curtis distances remained significantly different between the PD and HC cohorts after controlling for the confounding factors of age, sex, BSS, constipation and energy intake from diet (not shown).

#### Exploration of Composition Differences for Indicator Taxa

**FIGURE 2:**
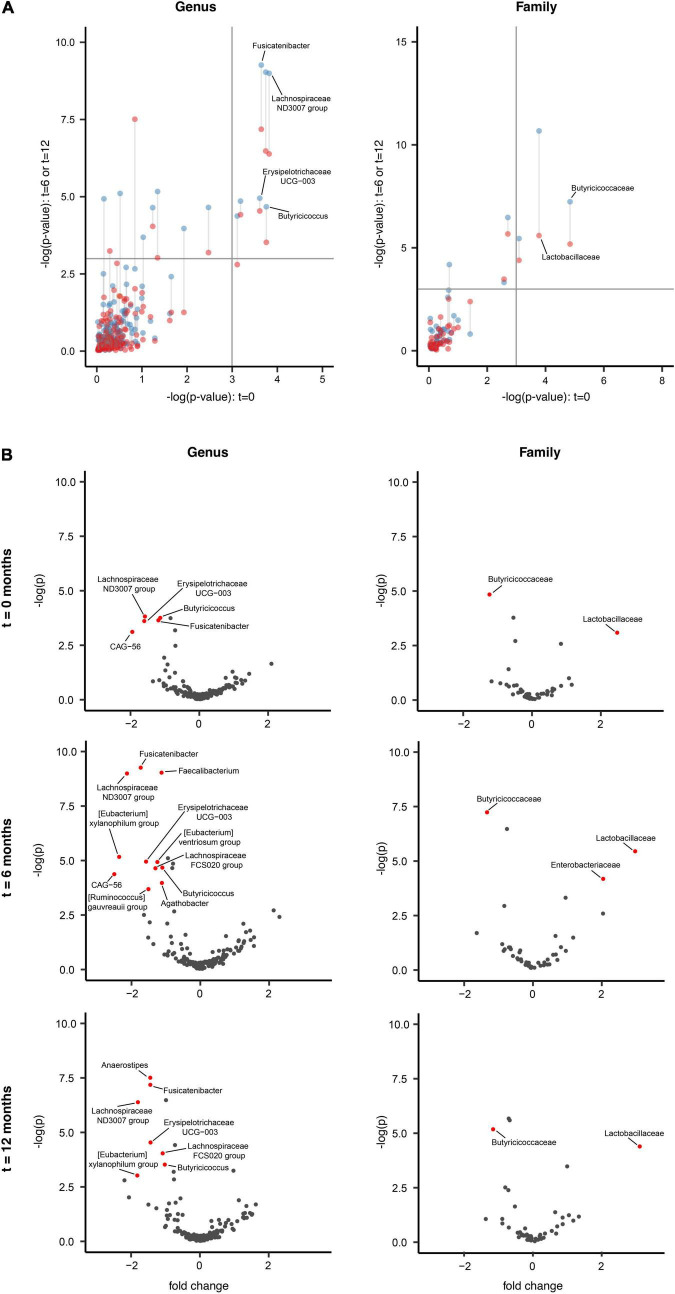
Longitudinal gut microbiota compositional changes in the Parkinson’s disease and household control cohorts. **(A)** ALDEx2 model-informed scatter plots identifying longitudinal GM differences between the PD (*n* = 74) and HC (*n* = 74) over the three *t* = 0 (*x*-axis), 6 (blue color; *y*-axis), 12 months (red color; *y*-axis) intervals, across two taxonomic levels of genus and family (*p* < 0.01). The upper right quadrant of the panel highlights persisting indicator taxa differences between PD and HC cohorts across all three-time intervals. Six taxa were consistently differentially abundant between the PD and HC groups across all three time points, which included underrepresentation of *Butyricicoccus, Fusicatenibacter, Lachnospiraceae ND3007 group* and *Erysipelotrichaceae UCG-003* in PD compared to the HC at the genus level, whilst an overrepresentation of *Lactobacillaceae* and underrepresentation of *Butyricicoccaceae* at the family taxonomic level. **(B)** Volcano plots representing abundance differences (fold change) of different taxa between HC and PD patients at *t* = 0, *t* = 6, and *t* = 12 months. Statistically significant [–log(p) > 3; fold change > ± 1.3] compositional differences at the genus and family levels (represented by red dots) were apparent and indicative of a PD-related GM composition. With regards to PD patients at the (i) genus taxonomic level and *t* = 0 interval, there was statistically significant underrepresentation of *Butyricicoccus*, *Fusicatenibacter*, *Lachnospiraceae ND3007 group, Erysipelotrichaceae UCG-003* and *Firmicutes bacterium CAG-56*. At the *t* = 6 interval an underrepresentation of *Butyricicoccus*, *Fusicatenibacter*, *Lachnospiraceae ND3007 group, Erysipelotrichaceae UCG-003, Faecalibacterium, [Eubacterium] xylanophilum group, [Eubacterium] ventriosum group, Lachnospiraceae FCS020 group, [Ruminococcus] gauvreauii group, Agathobacter*, and *Firmicutes bacterium CAG-*56. At the *t* = 12 interval an underrepresentation of *Butyricicoccus*, *Fusicatenibacter*, *Lachnospiraceae ND3007 group, Erysipelotrichaceae UCG-003, Anaerostipes, Lachnospiraceae FCS020 group*, and *[Eubacterium] xylanophilum group*. (ii) Family taxonomic level, at the *t* = 0 and *t* = 12 intervals, an overrepresentation of *Lactobacillaceae* and underrepresentation *Butyricicoccaceae*, whilst at the *t* = 6 interval an overrepresentation of *Enterobacteriaceae* and *Lactobacillaceae* and underrepresentation *Butyricicoccaceae* was seen.

The most authentic comparison was completed at ASV level, being the finest level of resolution possible. However, a problem with ASV is that it introduces noise, due to distinct ASVs (strains/species) having equivalent functional significance to gut ecosystem functions or PD, but non-equivalent distribution in the sample set. Thereby the lower ubiquity of ASVs lowers the statistical power for analysis. To address this issue, the analysis was repeated at higher taxonomic ranks, whereby “lumping” ASV’s resulted in taxa with greater ubiquity and thus higher power to detect significant associations. There is an assumption that the ASV’s collected into a higher taxon (genus, family, or order) still share meaningful functional attributes with respect to disease. Accordingly, comparison of the PD and HC cohorts at baseline, 6 and 12 months showed significant compositional differences at the genus, family and order levels across time intervals, indicative of PD-specific GM profiles (*p* < 0.01). The most differentially abundant taxa in PD patients are highlighted in both Figures 2A,B. When comparing PD with HC, we observed a persistent underrepresentation of the genera *Butyricicoccus, Fusicatenibacter, Lachnospiraceae ND3007 group* and *Erysipelotrichaceae UCG-003*, as well as an overrepresentation of *Lactobacillaceae* and underrepresentation of *Butyricicoccaceae* at the family level across all three-time intervals, signifying a persistently altered GM in PD patients. Subsequent PICRUSt2 analysis was completed to assess if these distinct taxa may have shared any attributes of potential relevance to PD, although no clearly supportive differences in pathways implicated in SCFA metabolism between the PD and HC groups were identified.

### The Influence of Parkinson’s Disease Progression Upon the Gut Microbiota

**FIGURE 3:**
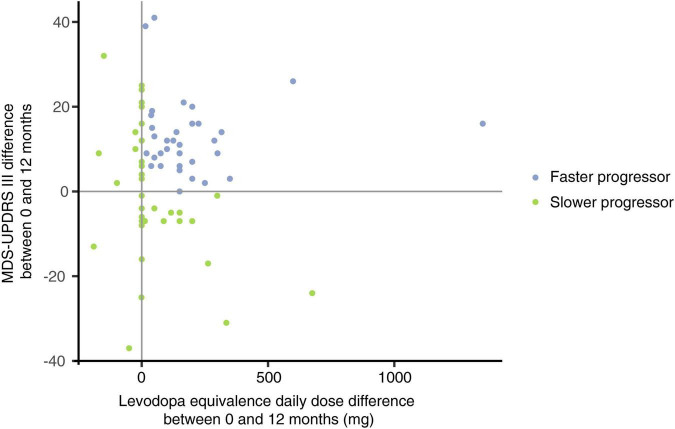
Characterization of faster and slower progressing Parkinson’s disease (PD) patients according to differential LED and MDS-UPDRS-III scores over 12 months. Faster progressing PD patients (blue; *n* = 34) and slower progressing patients (green; *n* = 40) were defined by the differential change in LED (*x*-axis) and MDS-UPDRS-III (*y*-axis) scores over the 12 months sampling period. A net increase in both LED and MDS-UPDRS-III scores over the 12 months was required to fulfill classification as a faster progressor, whilst individuals with either reduced or unchanged LED and/or MDS-UPDRS-III scores over the 12 months were considered slower progressors. LED and MDS-UPDRS-III scores were chosen as the most objective clinician-derived surrogate markers of PD progression from the obtained clinical data.

**FIGURE 4:**
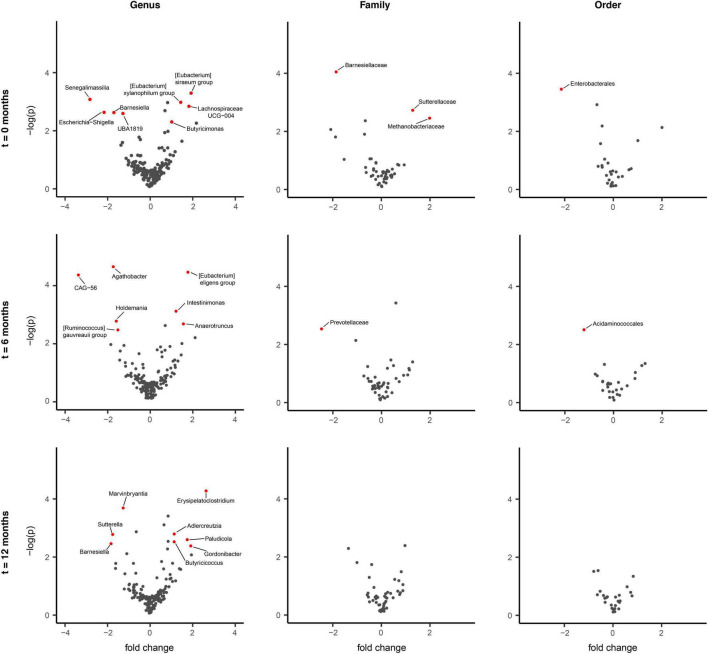
Differentially abundant taxa between faster and slower progressing Parkinson’s disease (PD) patients. ALDEx2 model-informed volcano plots identifying longitudinal gut microbiome (GM) differences between faster progressing (*n* = 34) and slower progressing (*n* = 40) PD patients over the three (*t* = 0, 6, 12 months) intervals. Comparing these two PD sub-cohorts revealed no statistically significant difference in GM composition across genus, family and order levels, persisting over the study duration. However, discrete microbiota differences were observed for each taxonomic level across each of the time intervals individually (*p* < 0.01 genus, <0.01 family, 0.02 order). *Barnesiella* appeared to be underrepresented in faster progressing PD patients at two-time intervals, *t* = 0 and *t* = 12 months. This was accompanied by an underrepresentation of *Barnesiellaceae* in faster progressing PD patients at the *t* = 0 interval.

**FIGURE 5:**
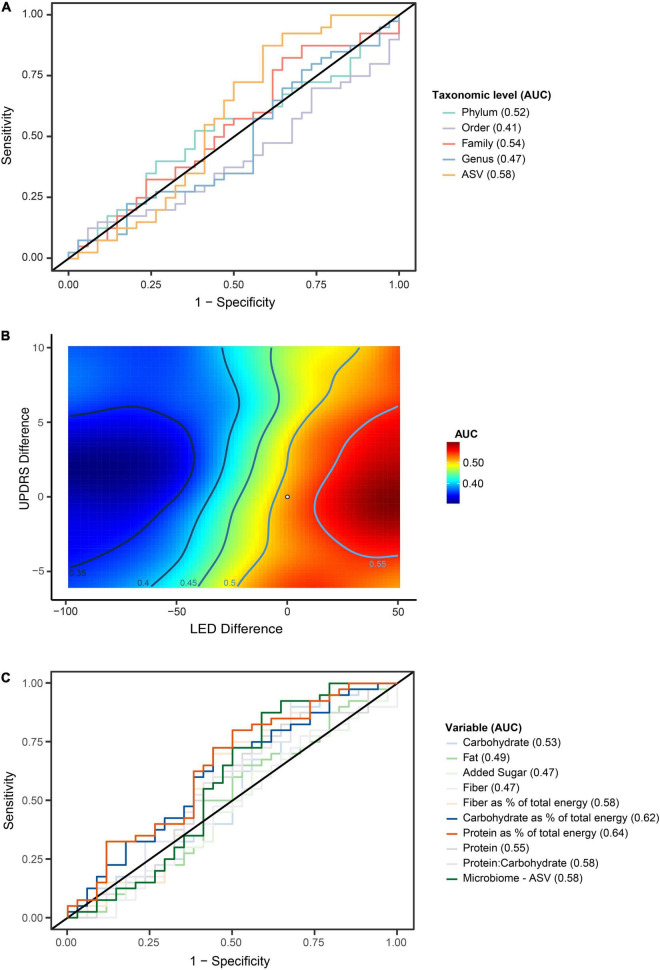
Random Forrest modeling demonstrates capacity to evaluate the gut microbiome to differentiate between faster and slower progressing Parkinson’s disease (PD) patients. **(A)** Combined central log transformation with support vector machine modeling utilizing the entire gut microbiome data alone at phylum, order, family, genus and Amplicon Specific Variant (ASV) taxonomic levels to differentiate between faster and slower progressing PD patients. The best predictability was observed at the ASV taxonomic level with an Area Under the Curve (AUC) of 0.58. **(B)** Variation of the detection thresholds for levodopa equivalence dose and difference in MDS-UPDRS-III score differences was explored in an effort to optimize the AUC. A mild increase of the LED threshold was able to produce a higher AUC, with the region around the optimized cut-off (indicated by a dot), was relatively stable with AUC ranging between (0.55–0.58). **(C)** Random Forest modeling incorporating microbiome and clinical nutritional data (two-step model) was performed to improve differentiation in predicting faster disease progression at ASV taxonomic level. Testing of various nutritional variables with the model showed that incorporation of patient protein intake as a percentage of total energy provided increased prediction for faster progressing patients, with an AUC of 0.64.

To evaluate for potential GM compositional changes in PD in response to disease progression, the longitudinal PD cohort of *n* = 74 was divided into faster *n* = 34 and slower *n* = 40 progressing patients (Figure 3). We found evidence that faster versus slower progressing PD cohorts had different GM compositions, but these apparent differences weren’t consistently maintained over the three-time intervals (*p* < 0.01 genus, *p* < 0.01 family, *p* = 0.02 order, *p* = 0.04 phylum) (Figure 4). The genus *Barnesiella* appeared to be underrepresented in faster progressing PD patients at two time intervals *t* = 0 and *t* = 12 months. This was further accompanied by an underrepresentation of the family *Barnesiellaceae* in faster progressing PD patients at baseline (Figure 4). A search for potential confounders identified differing BSS scores [faster progressors 2.5 (*SD* 0.9) and slower progressors 3.1 (*SD* 0.9)] between the two groups. These GM changes could be attributable to multiple environmental factors, including change in physiological state and diet over time. To address this, machine learning algorithms utilizing combined central log transformation with support vector machine modeling were applied in conjunction with latent class to examine the predictive utility of the gut microbiota in defining PD progression. Examining the GM data alone, different levels of taxonomic resolution were tested, identifying that the best prediction of disease progression was provided by the ASV level, with an AUC of 0.58 (Figure 5A). It is important to note that while varying the detection thresholds of the MDS-UPDRS-III scores and LED will result in different AUC estimates (Figure 5B), the region around our chosen cut-off was relatively stable with AUC ranges between (0.55 – 0.58). Lastly, we developed a two-stage model that integrated patient nutritional data. In particular, the inclusion of protein contribution to total energy intake in the model improved the predictive power to an AUC of 0.64 from an AUC of 0.58 when considering the ASV taxonomic level alone (Figure 5C).

### Temporal Dynamics of the Gut Microbiome Following Initiation of Deep Brain Stimulation and Levodopa-Carbidopa Intestinal Gel Therapies

#### Relative Abundance, Diversity and Search for Indicator Species of Therapy Type

**FIGURE 6:**
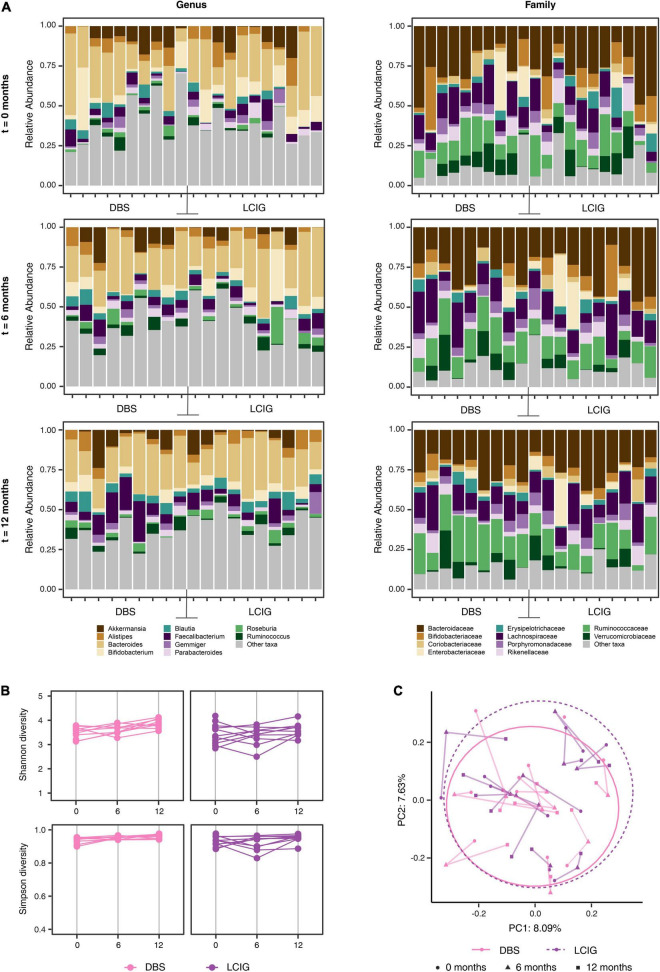
Longer-term device-assisted therapies influence on the gut microbiome. **(A)** Individual device-assisted therapy (DAT) Parkinson’s disease (PD) patient (*n* = 9 DBS and *n* = 10 LCIG) mean relative abundance differences at the genus and family taxonomic levels at 0, 6, and 12 month intervals, showed statistical significance between the two groups across the study (PERMANOVA *p* < 0.01 genus, <0.01 family). **(B)** Alpha diversity (Shannon and Simpson diversity) at the Amplicon Specific Variant (ASV) level for Deep Brain Stimulation (DBS) and Levodopa-Carbidopa Intestinal Gel (LCIG) DAT patients, across 0, 6, and 12 month intervals showed no significant alpha-diversity differences between the groups over the study period (*p* = 0.06 Shannon, *p* = 0.654 Simpson). **(C)** Beta-diversity explored by Principal Component Analysis with Bray–Curtis ordination at the ASV level revealed statistically significant differences in beta diversity between the two groups (*n* = 9 DBS and *n* = 10 LCIG) across the study period (PERMANOVA *p* < 0.01).

**FIGURE 7:**
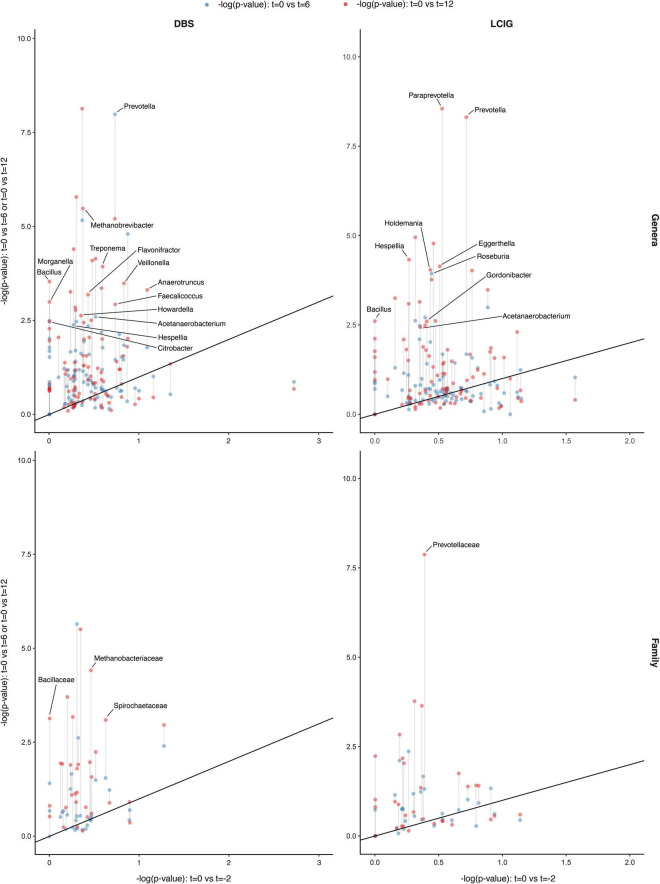
Gut microbiome compositional differences in response to the initiation and continuation of Deep Brain Stimulation (DBS) and Levodopa-Carbidopa Intestinal gel (LCIG) therapies. A center log transformed ALDEx2 model identified differentially represented taxa in response to the effect of device-assisted therapy (DAT) treatment initiation and continuation, [intervals *t* = 0 vs. *t* = –2 (*x*-axis), *t* = 0 vs. *t* = 6 and *t* = 0 vs. *t* = 12 (*y*-axis)] were compared. Results across two taxonomic ranks (genera and family) for DBS and LCIG participants showed that the most differentially abundant taxa after the initiation of DBS therapy (*t* = 0 to *t* = 6) included overrepresentation of *Prevotella* at the genus level. Whilst across the (*t* = 0 to *t* = 12) interval (1) overrepresentation of *Methanobacteriaceae, Bacillaceae* and *Spirochaetaceae* at the family level. (2) Underrepresentation of *Hespellia, Acetanaerobacterium, Anaerotruncus, Howardella* and *Flavonifractor*, whilst and overrepresentation *Prevotella*, *Methanobrevibacter, Treponema, Bacillus, Veillonella, Citrobacter, Faecalicoccus*, and *Morganella* at the genus level. Whilst in response to LCIG therapy initiation and continuation over the (*t* = 0 to *t* = 6) interval included overrepresentation of *Roseburia* at the genus level. Whereas (1) overrepresentation of *Prevotellaceae* at the family level, (2) underrepresentation of *Hespellia, Eggerthella, Holdemania, Gordonibacter*, and *Acetanaerobacterium* and (3) overrepresentation of *Prevotella* and *Bacillus* at the genus level were apparent across the (*t* = 0 to *t* = 12) interval. Within the DBS cohort, a persisting overrepresentation of *Prevotella* was noted over both 6 and 12 months intervals since DBS initiation, whilst the LCIG cohort did not show a definitive persistent taxa change across both 6 and 12 months intervals. Although a notable trend of overrepresentation of *Roseburia* was seen during the (*t* = 0 to *t* = 12) interval, although not quite reaching statistical significance, *p* = 0.051.

Consistent with the longitudinal PD and HC cohorts, the mean relative abundance of bacterial taxa were generally similar between DBS and LCIG groups across all sampling time points, although statistically significant mean relative abundance differences were seen (PERMANOVA; *p* < 0.01 genus, *p* < 0.01 at the family level) (Figure 6A). Alpha diversity metrics of microbial communities from all PD DAT patients were comparable across the sampling period (ANOVA; *p* = 0.06 Shannon, *p* = 0.654 Simpson) (Figure 6B). The effects of beta diversity change for the DAT cohort over the 12 months period were considered at the ASV level using Bray–Curtis ordination, with a statistically significant difference noted between the groups (PERMANOVA; *p* < 0.01) (Figure 6C). Further exploring potential GM compositional differences in response to the initiation and continuation of DBS or LCIG therapies, a center log transformed ALDEx2 model was used to identify differentially represented taxa. The effect of treatment continuation was compared between three intervals, (*t* = 0 vs. *t* = –2), (*t* = 0 vs. *t* = 6), and (*t* = 0 vs. *t* = 12), with results shown at two levels of taxonomic resolution (Figure 7). Each point in the plot represents a taxon with the *x*-axis showing changes in *t* = 0 vs. *t* = –2 and the *y*-axis representing the change from *t* = 0 vs. *t* = 6 to *t* = 0 vs. *t* = 12.

The most differentially abundant taxa after the initiation of DBS therapy (*t* = 0 to *t* = 6) included overrepresentation of *Prevotella* at the genus level. Whilst across the (*t* = 0 to *t* = 12) interval, we found: (1) overrepresentation of *Euryarchaeota* and *Spirochaetes* at the phylum level; (2) overrepresentation of *Bacillales*, *Methanobacteriales*, and *Spirochaetales* at the order level; (3) overrepresentation of *Methanobacteriaceae, Bacillaceae*, and *Spirochaetaceae* at the family level; (4) underrepresentation of *Hespellia, Acetanaerobacterium, Anaerotruncus, Howardella*, and *Flavonifractor*, with an overrepresentation of *Prevotella*, *Methanobrevibacter, Treponema, Bacillus, Veillonella, Citrobacter, Faecalicoccus*, and *Morganella* observed at the genus level (Figure 7).

The most differentially abundant taxa after the initiation of LCIG therapy included overrepresentation of *Roseburia* at the genus level over the (*t* = 0 to *t* = 6) interval. Whilst across the (*t* = 0 to *t* = 12) interval, we found: (1) overrepresentation of *Bacillales* at the order level; (2) overrepresentation of *Prevotellaceae* at the family level; (3) underrepresentation of *Hespellia, Eggerthella, Holdemania, Gordonibacter*, and *Acetanaerobacterium* and overrepresentation of *Prevotella* and *Bacillus* at the genus level (Figure 7).

Within the DBS cohort, a persistent over representation of *Prevotella* was noted over both 6 and 12 months timepoints following DBS initiation, whilst the LCIG cohort did not show a clearly persistent taxa change across both 6 and 12 months intervals. However, a trend of overrepresentation of *Roseburia* was seen for the (*t* = 0 to *t* = 12) interval, although not quite reaching statistical significance, *p* = 0.051. Between the DBS and LCIG cohorts, only an overrepresentation of the genus *Bacillus* over the (*t* = 0 to *t* = 12) interval was noted, implying individualized effects from each DAT on the GM.

## Discussion

We conducted a longitudinal study of microbiome dynamics over a 12 month period, in a well characterized PD cohort with environment-matched HCs and made three major observations. Firstly, our study showed a persistent difference in representation of multiple indicator taxa between PD patients and HCs over time. Secondly, the use of a latent class modeling approach supported the potential for predictive tests of PD progression, if diet is also taken into account. Thirdly, GM impacts of initiating DBS and LCIG differed in response to shorter and longer term DAT exposure.

The three existing longitudinal studies have reported on a variety of influences that alter the GM in PD patients (Minato et al., 2017; Aho et al., 2019; Jin et al., 2019; Hegelmaier et al., 2020), although with variations in study design, meaningful comparison or identification of emerging patterns is difficult. Whilst earlier longitudinal studies consistently identify indicator taxa differences between PD and HCs or with PD interventions, differences in progression scores, metadata on diet, PD history or reference point for defining microbiome change and inconsistent controlling factors that endorse sustained microbiota differences of sufficient predictive power for clinical application. The present study was notably different from previous longitudinal PD GM studies, where despite a shorter study duration, a broader range of potential indicator taxa for microbiome differences between PD patients and HCs was apparent, several of which have been reported in other studies (Nishiwaki et al., 2020; Romano et al., 2021). Specifically, underrepresentation of the genera *Butyricicoccus, Fusicatenibacter, Lachnospiraceae ND3007 group* and *Erysipelotrichaceae UCG-003* in PD patients compared to the HCs, persisted across all three time intervals. This supports our hypothesis that disease-associated GM profiles exist in PD patients, and that exploration of the GM as a biomarker for PD progression is warranted.

The SCFAs, butyrate, propionate and acetate, are the major end products of bacterial fermentation of dietary fiber and are considered to have an important role in maintaining the integrity of the colonic epithelium (Romano et al., 2021). All three SCFAs contribute to signaling functions *via* G-protein-coupled receptors that can impact metabolic and immune functions (Andreu et al., 2021). Acetate is the major SCFA and is produced by multiple bacteria. Its production potential is thought to be relatively insensitive to community change but does change with amount of microbial substrate (dietary fiber) (Andreu et al., 2021). Propionate and butyrate are produced by fewer groups of bacteria and their levels are more prone to change with community structure (Andreu et al., 2021). Butyrate is of particular interest as it has special significance for gut barrier functions and butyrogenesis is predominantly contributed by taxa that have been reported to be of lower relative abundance in PD (Andreu et al., 2021). The underrepresentation of microbiota with butyrogenic potential in PD may support a mechanistic pathway promoting gut leakiness and pro-inflammation that can facilitate α-syn aggregation and spread, the degeneration of the gut-brain-axis and the progression of PD. Comparison of the indicator taxa differences identified in this study with earlier studies supports an emerging pattern of underrepresented genera from the SCFA-producing *Lachnospiraceae* family in PD cohorts compared to HCs (Weis et al., 2019; Wallen et al., 2020; Romano et al., 2021). The underrepresented genera *Fusicatenibacter, Lachnospiraceae ND3007 group* and *Erysipelotrichaceae UCG-003* identified here, are all part of the *Lachnospiraceae* family and are abundant in the digestive tracts of many mammals. They offer protection from colon cancer due to the butyrogenic producing capacity of many species within the family, which is an important metabolite for both microbial and host epithelial cell growth and function (Meehan and Beiko, 2014). Consistently, *Fusicatenibacter* have anti-inflammatory, neuroprotective and other beneficial effects on the epithelial barrier in PD (Weis et al., 2019). Furthermore, *Butyricicoccus* (Eeckhaut et al., 2013) are butyrate-producing bacteria associated with decreased intestinal myeloperoxidase, tumor necrosis factor-α and interleukin-12 levels in inflammatory bowel disease rat models, contributing to reduced pro-inflammatory potential in the gut microenvironment (Eeckhaut et al., 2013). Future studies should continue to validate the theory of reduced SCFAs in PD by metabolomics as well as other multi-omics approaches (Tan et al., 2021).

Due to increasing recognition of prodromal PD symptoms, namely GI dysfunction and constipation, emerging research interests have shown that alterations in GM composition compared to HC have been identified in prodromal markers and early PD, particularly in REM Sleep Behavior Disorder (Heintz-Buschart et al., 2018; Heinzel et al., 2021). Recognition of early alterations in GM composition in PD have further driven inquest in utilizing the GM as a potential biomarker to assist clinicians in informing disease progression. Defining PD progression can be a challenging clinical undertaking, particularly due to variability of patient motor responses during on and off medication cycles, as well as other environmental confounding factors. Further, prodromal and clinical PD may be heterogeneous in its presentation and has been proposed to be classified in various conceptual subtypes, reflective of differing patterns of spatial and temporal progression throughout the peripheral and central nervous systems (Berg et al., 2021). Individuals with REM Sleep Behavior Disorder have been considered to exhibit a distinct body-first subtype, rather than brain-first subtype of PD, due to indicative patterns of α-syn pathology spread (Berg et al., 2021). Improved characterization of different PD subtypes in early disease may provide unique insights to better understand PD progression *via* the integration of novel biomarkers. Exploring the utility of the GM as a useful indicator for PD progression certainly holds great interest and appeal (Qian et al., 2020). Such a marker could assist clinicians in better tailoring appropriate therapy selection, as well as gauging disease response and related complications, such as early motor fluctuations. Our progression analysis found statistically significant taxa differences between faster and slower progressing PD patients across the three timepoints. However, no clear indicator taxa persisted across all three time intervals, suggesting that the observed patterns were more reflective of interpersonal differences rather than disease progression. Nevertheless, a trend of underrepresentation of *Barnesiella* in faster progressing PD patients at two time intervals (*t* = 0 and *t* = 12 months) was observed, warranting further evaluation in a larger cohort and over a longer sampling period. In contrast, *Barnesiella* along with three other indicator taxa were reported to be overrepresented in PD patients with mild-cognitive impairment, although this data originated form a small cohort of 13 patients, with no mention of disease severity or progression (Ren et al., 2020). *Barnesiella*, is known for its favorable antibiotic-resistant properties that prevents pathogenic vancomycin-resistant enterococci from colonizing the gut, in addition to enhancing the effectiveness of immunomodulatory therapy for certain types of cancer (Ubeda et al., 2013). The potential mechanisms underlying its influence on PD pathophysiology, particularly PD progression, are presently unknown. Similar difficulty in characterizing persistently differentially abundant taxa in faster progressing PD patients was encountered by Aho et al. (2019), who examined GM profiles to characterize progression in their cohort of 41 stable and 15 progressing PD patients, noting only an overrepresentation of *Prevotella* in more stable patients. The small sample sizes in these two studies, as well as relatively short duration of follow up, likely limit the ability to define an accurate GM indicator of PD progression.

We were able to demonstrate that variation in the detection thresholds of the net difference in MDS-UPDRS-III and LED scores to classify faster and slower progressing patients had an influence on predictive modeling of disease progression. Setting a detection threshold of mildly increasing LED difference appeared to result in a higher AUC for the prediction of faster progressing PD patients. This was further supported by the newly developed two-stage model, which showed that the incorporation of nutritional data, specifically protein contribution to total energy intake, was able to stratify the cohort into a subpopulation where the association between GM and PD progression was improved. The interpretability of why certain nutritional markers assist in characterizing disease progression is poorly understood and warrants further study (Xu et al., 2021). The recognized preference for increased carbohydrate intake in PD (Palavra et al., 2021) may lead to potential restrictive consumption of protein and its inadvertent contribution to total energy, which could also be further influenced by patient preference to minimize potential levodopa malabsorption, particularly with higher and frequent regiments of levodopa in more advanced disease. Additionally, improved and more quantifiable measures of disease progression need to be developed to increase the likelihood of characterizing disease progression. The progression modeling studied here was in a heterogenous PD population of varying disease severities and needs to be replicated in more restricted groups, namely the prodromal, preclinical, and also more advanced populations, to better guide applicability of when the most useful predictive capacity for such models exists. Earlier characterization of faster progressing patients is integral to providing optimal clinical management aimed to improve patient QoL and reduce motor-fluctuations. Often this translates to earlier integration of adjuvant therapies, including DAT, as well as multidisciplinary care. Ultimately, larger longitudinal cohorts with longer sampling periods and more robust criterion for defining faster and slower progressing patients are necessary to refine the utility of the GM if progression of PD is to be followed in this manner.

Longitudinal interrogation of the DAT cohorts over a 1 year period showed no persistent over or underrepresentation of microbiota at any taxonomic level, which is consistent with our previously reported short-term (*t* = 2 and *t* = 4 weeks) interrogation intervals following DAT initiation (Lubomski et al., 2021c). This may be due to differing gut physiological and motility adaptations that develop in response to chronic exposure to DATs, including modifications in constipation and upper gastrointestinal dysfunction severity, as well as other environmental factors. Although, no significant changes over time could be appreciated by our self-reported GI questionnaires in both groups. It is hypothesized that the influences of these DATs on the GM are likely bidirectional. Other potential influences include more distant exposure from intravenous Cephazolin antibiotics, which were mandated for all DBS patients at *t* = 0, to reduce risk of hardware infection, as well as mild acidification of the gut due to LCIG use (approximately pH 6). Alternatively, these DATs may manifest with more gradual influences on the gut microbiota, which can be better appreciated with the extended longitudinal follow up. The persisting overrepresentation of *Prevotella* at the 6 and 12 months intervals following DBS initiation, and the trend suggestive of overrepresentation of *Roseburia* at 6 and 12 months in LCIG participants, requires validation in larger cohorts of DAT patients, over longer periods, as overrepresentation of *Prevotella* has been suggested to reflect more stable and slower progressing PD patients (Aho et al., 2019). Overrepresentation of *Prevotella* in this study could reflect the typically observed improvement in PD control many patients experience after DBS initiation, likely due to secondary environmental, lifestyle and nutritional changes often adopted after improved PD control. *Prevotella* species are frequently reported with controversy in the literature, likely due to the high genetic variety, which makes it difficult to predict their function (Ley, 2016) and therefore their potential beneficial or detrimental influence on gut health and anti-inflammatory properties (Ley, 2016). Moreover, the observed trend toward overrepresentation of *Roseburia* in PD patients continuing LCIG therapy is particularly noteworthy, given their favorable SCFA-producing activity (Parada Venegas et al., 2019) and association with weight loss and reduced glucose intolerance in mice (Ryan et al., 2014). These findings are also increasingly recognized in patients enduring chronic LCIG use (Fabbri et al., 2019). Interestingly, the short-term changes in taxa abundance 2 and 4 weeks after the initiation of DAT (DBS and LCIG) reported previously (Lubomski et al., 2021c), were inconsistent with those changes observed at later timepoints (6 and 12 months) in this study. This suggests either a differing physiological response or a possible DAT-mediated influence on the GM that differs depending on duration of therapy exposure. Alternatively, it may indicate a refractory GM response to the introduction of a new stimuli (i.e., DAT) that is short-lived and then more considerable remodeling of the GM occurs over time. Ultimately, PD patients requiring DAT are likely to have altered GM compositions prior to DAT initiation due to multiple factors, including older age, longer disease duration, motor fluctuation, increased GI dysfunction (namely constipation) and are likely to require more PD medication classes and increased dosages, when compared to those who don’t require DAT.

Several limitations were considered in our study, the non-optimally matched PD and HC groups for age and sex, due to spousal recruitment, may have resulted in a potential confounder in the comparative GM analysis, as age, sex, BMI, diet, and various medications have been shown to influence the GM (David et al., 2014; Hill-Burns et al., 2017; Aho et al., 2019; Baldini et al., 2020). The utilization of cohabitants or spousal controls is generally more suitable to adjust for geographic and environmental confounders, although differences in age and sex distributions as noted in our and many former PD GM studies, as well as other methodological inconsistencies across the studies, may account for the heterogeneity in reported GM profile differences. It is also often challenging to directly compare indicator taxa between GM studies, due to the updating of taxa reference datasets across varied bioinformatic pipelines, which can result in inconsistencies of taxa classification amongst studies.

Our study was unable to address other potential confounding factors, including other comorbidities and other non-PD medication effects. Information on medication use for GI dysfunction (e.g., laxatives, anti-diarrhoea medication and reflux medication), was not available for consideration in our analysis, even though they are important modulators, as well as GI tract medical diagnosis (e.g., inflammatory bowel disease, inflammatory bowel syndrome, coeliac disease, previous gut surgery or gastrointestinal tumors), as reported earlier (Lubomski et al., 2020b, 2021c). Whether these covariates alter the PD-specific GM profiles is yet to be established, although we have demonstrated that PD patients have increased symptoms of GI dysfunction (Lubomski et al., 2020b). The variability of the differential abundance of taxa between prior PD GM studies is heavily influenced by differing methodological considerations (sample sizes, sampling and storage techniques, extraction and sequencing protocols, bioinformatic approaches between the studies, geographic locale and nutritional intake) and may certainly be a key determinant of the taxa variability reported between PD GM studies. The classification of faster and slower progressing PD patients was challenging due to the need to obtain consistently comparable motor severity scores across all sampled intervals. Despite methodological considerations made to assess patient motor severity during their “on” state with accompanying contemporaneous account of all PD medications to inform LED at each review, instances including suboptimal medication compliance, partial responses or dose failure may have impaired best “on” state assessment. Furthermore, the follow up interval duration also posed difficulties for adequately defining faster progressing patients, due to the potential time lag for a clinician-guided LED adjustment, to compensate for motor progression. This may be due to patients undergoing infrequent clinical reviews, resulting in a delayed opportunity to optimize their PD medication, meaning such patients were inadvertently not captured in the faster progressing group, due to lack of LED increase. Likewise, patients with a net increase in their LED difference over the 12 months but without an increased MDS-UPDRS-III score, were not considered faster progressors due to potential underestimation of their true motor severity by sub-optimal definition of the “on” state. Accordingly, we decided the most appropriate measure to categorize faster from slower progressing patients was based on a synchronous increase in the difference of MDS-UPDRS-III and LED scores over the 12 months interval. It is also likely that differences in age and disease stages may account for changes in our progression analysis, with faster progressing patients possibly being younger and at earlier disease stages. Our findings should be interpreted in light of their limitations, including the self-reporting nature of the data and potential selection bias of the study population being drawn from specialist PD clinics within a single metropolitan area. This is important as previous studies from Australia have shown PD patients from regional areas are comparatively older with an older age of diagnosis (Lubomski et al., 2013, 2014, 2015).

## Conclusion

Our longitudinal cohorts of existing therapy PD patients and those who recently initiated DATs present unique perspectives in a PD GM literature primarily informed from cross-sectional observations. Both groups show persistent and significant alterations of gut microbiota abundances between PD patients and HCs, supporting the emerging trends of overrepresented proinflammatory and mucus degrading microbiota communities that may increase intestinal barrier permeability and cause gut leakiness. We have demonstrated that over 12 months, there was a persistent underrepresentation of known SCFA-producing bacteria, particularly those with a butyrogenic production potential in PD patients, supporting the hypothesis of abnormal maintenance of gut wall permeability and leakiness. The compositional GM differences between the PD and HC cohorts in our study were comparable to many other prior original studies, as well as highlighting novel taxa that require further validation as part of larger longitudinal studies and meta-analyses. Progression analysis utilizing the GM showed suggestive trends of underrepresented *Barnesiella* in faster progressing patients, with predictive modeling also suggestive that incorporation of nutritional data, specifically protein contribution to total energy intake, can improve predictive ability to identify faster progressing patients. Larger cohorts and lengthier follow-up intervals are needed to clearly define trends suggestive of altered microbial community abundances during disease progression. DBS and LCIG influences on the GM are likely complex and multifactorial, however individually highlight variable short-term and delayed influences to the GM in response to DAT initiation, which should also be examined as part of larger studies.

## Code Availability

For this study, no custom functionalities were coded to generate or process the dataset, whilst the R code available is available https://github.com/SydneyBioX/microbiome-PD. All used software and packages, their versions, relevant specification and parameters are stated in the “Materials and Methods” section in the paragraph “computational and statistical analyses.” More detailed information is available from the corresponding author on reasonable request.

## Data Availability Statement

The datasets presented in this study can be found in the online repository https://www.ncbi.nlm.nih.gov/bioproject/PRJNA808166.

## Ethics Statement

The studies involving human participants were reviewed and approved by the Northern Sydney Local Health District Human Research Ethics Committee and the North Shore Private Hospital Ethics Committee, HREC/18/HAWKE/109, NSPHEC 2018-LNR-009, respectively. The patients/participants provided their written informed consent to participate in this study.

## Author Contributions

ML conceived and designed the study, recruited and examined all participants, collected and analyzed the data, and drafted and reviewed the manuscript. XX analyzed the genomic and clinical data, and drafted and reviewed the manuscript. AH drafted and reviewed the manuscript. SM and JY analyzed the genomic data, and drafted and reviewed the manuscript. RD conceived and designed the study, generated the data, and drafted and reviewed the manuscript. CS conceived and designed the study, and drafted and reviewed the manuscript. All authors contributed to the article and approved the submitted version.

## Conflict of Interest

The authors declare that the research was conducted in the absence of any commercial or financial relationships that could be construed as a potential conflict of interest.

## Publisher’s Note

All claims expressed in this article are solely those of the authors and do not necessarily represent those of their affiliated organizations, or those of the publisher, the editors and the reviewers. Any product that may be evaluated in this article, or claim that may be made by its manufacturer, is not guaranteed or endorsed by the publisher.
