# Supplementary material for: The Gut Microbiome in Parkinson’s Disease: A Longitudinal Study of the Impacts on Disease Progression and the Use of Device-Assisted Therapies
Source: Front Aging Neurosci. 2022 May 17;14:875261. doi: 10.3389/fnagi.2022.875261 (PMC9152137; doi:10.3389/fnagi.2022.875261)
Supplement: Supplementary file 1 [file Data_Sheet_1.DOCX]

**The gut microbiome in Parkinson’s disease: a longitudinal study of the impacts on disease progression and the use of device-assisted therapies.**

**Supplementary Data**

**Figures:**


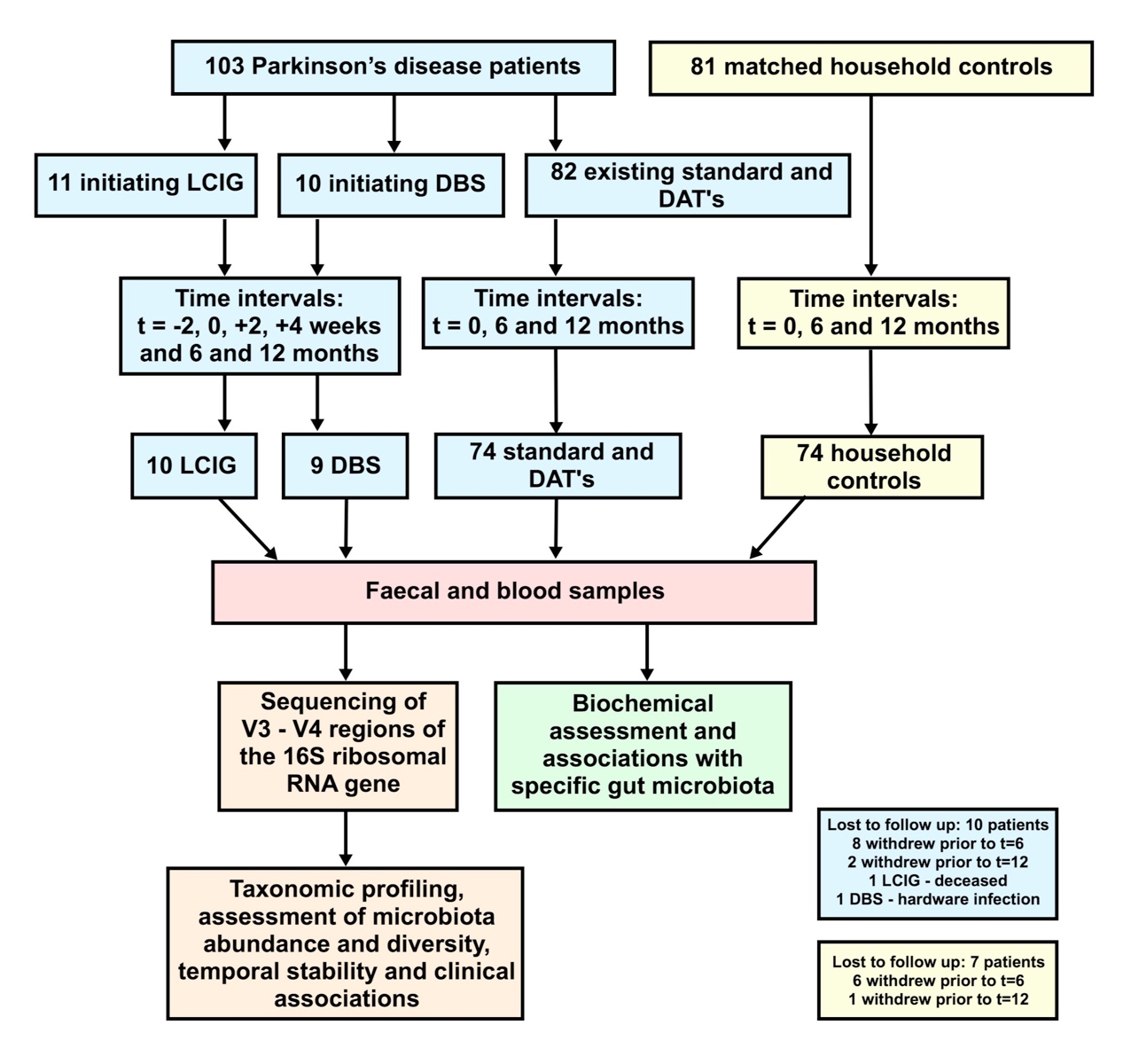


**Supplementary Figure 1: Recruitment and analysis of Parkinson’s disease (PD) and household control (HC) participants.** Diagram of enrolled PD and HC participants in this study. All participants provided biospecimens for 16S amplicon sequencing analysis of the gut microbiome at various timepoints and routine biochemical blood tests. Two cohorts are described: 1) 82 Parkinson’s disease patients continuing with their existing or established therapy and 81 matched spousal household controls were recruited. Analysis of the existing therapies cohort was completed as part of n=74 PD and n=74 HCs, to ensure all participants were represented across all the three time intervals. 2) Device-assisted therapies cohort of n=11 Levodopa-carbidopa intestinal gel (LCIG), and n=10 Deep Brain Stimulation (DBS) initiating patients were recruited. One LCIG patient passed away and one DBS patient developed a hardware infection and were excuded from the study. Final analysis was conducted on n=10 LCIG and n=9 DBS patients, to represent consistent clinical and microbiome data across all sampled time intervals. Following 16S sequencing, taxonomic profiling was completed to assess differences between groups and therapies, temporal stability and clinical associations with the gut microbiome.


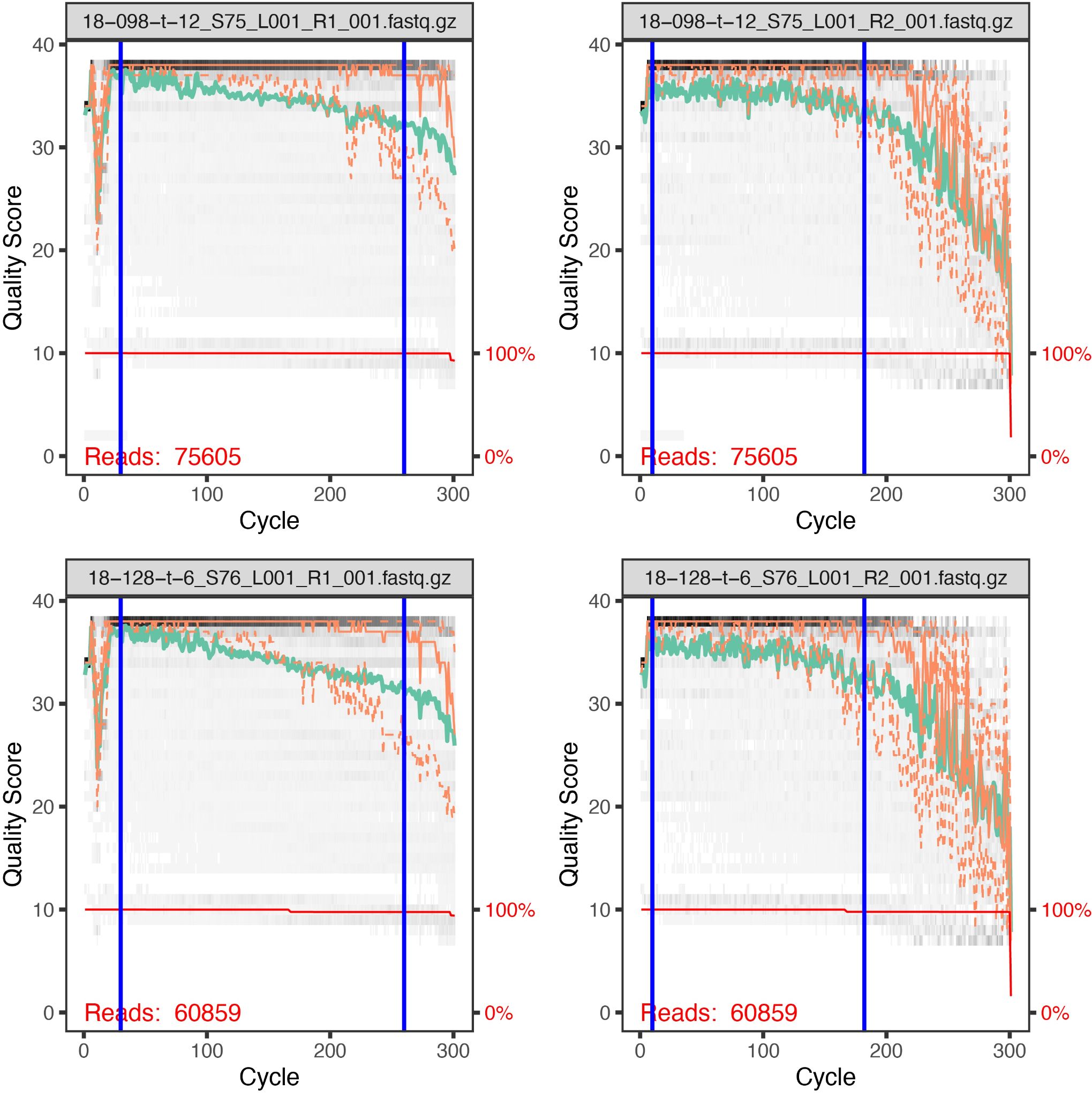


**Supplementary Figure 2:**Representative samples showing raw-paired sequencing read quality (R1 in the left column, R2 in the right column). Reads were trimmed at either end to give between 150 and 250 bases of sequence with read quality score >30. The total number of reads per sample is shown in red in the bottom left of each plot.


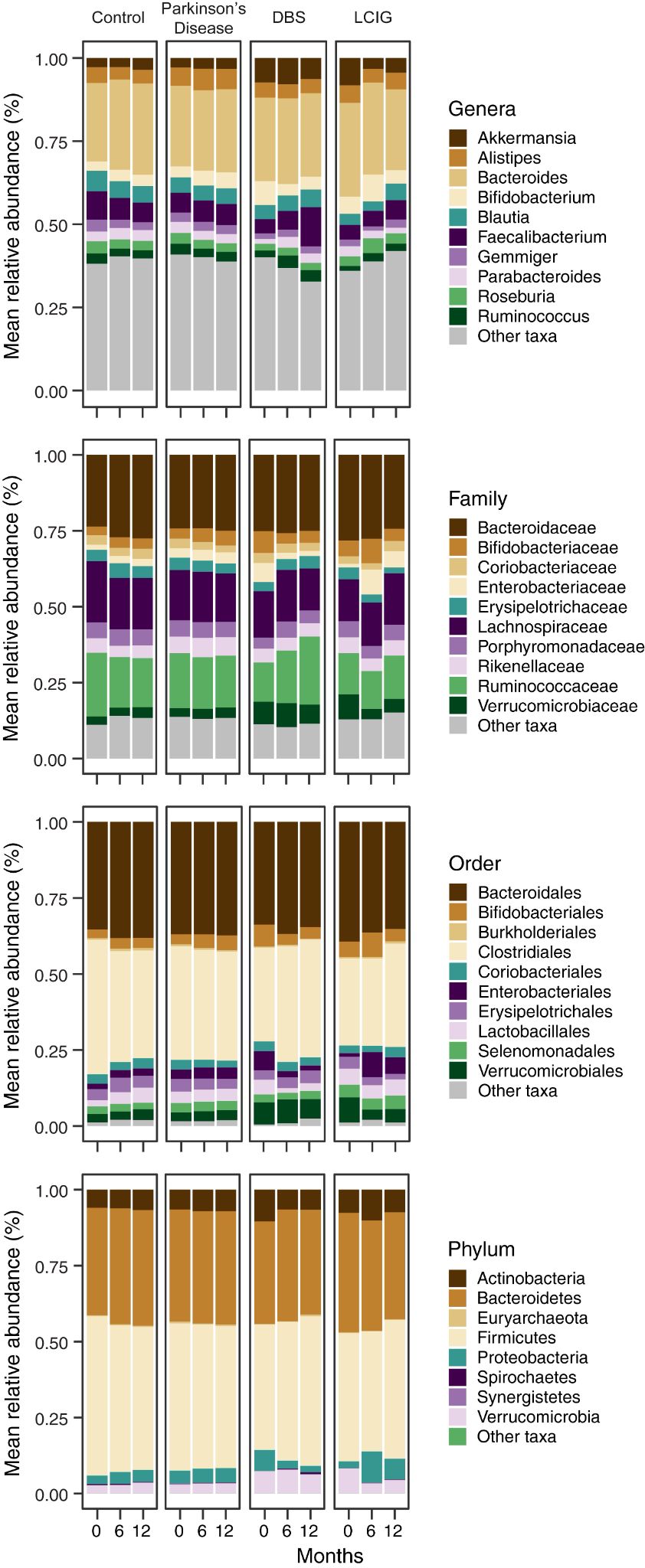


**Supplementary Figure 3: Mean relative abundance of gut microbiota representative of the two longitudinal cohorts of this study.** Relative abundance differences for the existing therapies longitudinal cohort of n=74 PD and n=74 HC participants, shown at genus, family, order and phylum taxonomic levels at 0, 6 and 12 month intervals demonstrated comparable mean relative abundances between the two groups (PERMANOVA; p<0.01 genus, p<0.01 family, p<0.01 order and p=0.03 phylum). In addition, the device-assisted therapies longitudinal cohort of n=9 DBS and n=10 LCIG PD patients, at 0, 6 and 12 month intervals, also show overall comparable, yet statistically significant, mean relative abundances at genus, family, order and phylum taxonomic levels (PERMANOVA p<0.01 genus, p<0.01 family, p=0.02 order and p=0.03 phylum levels).


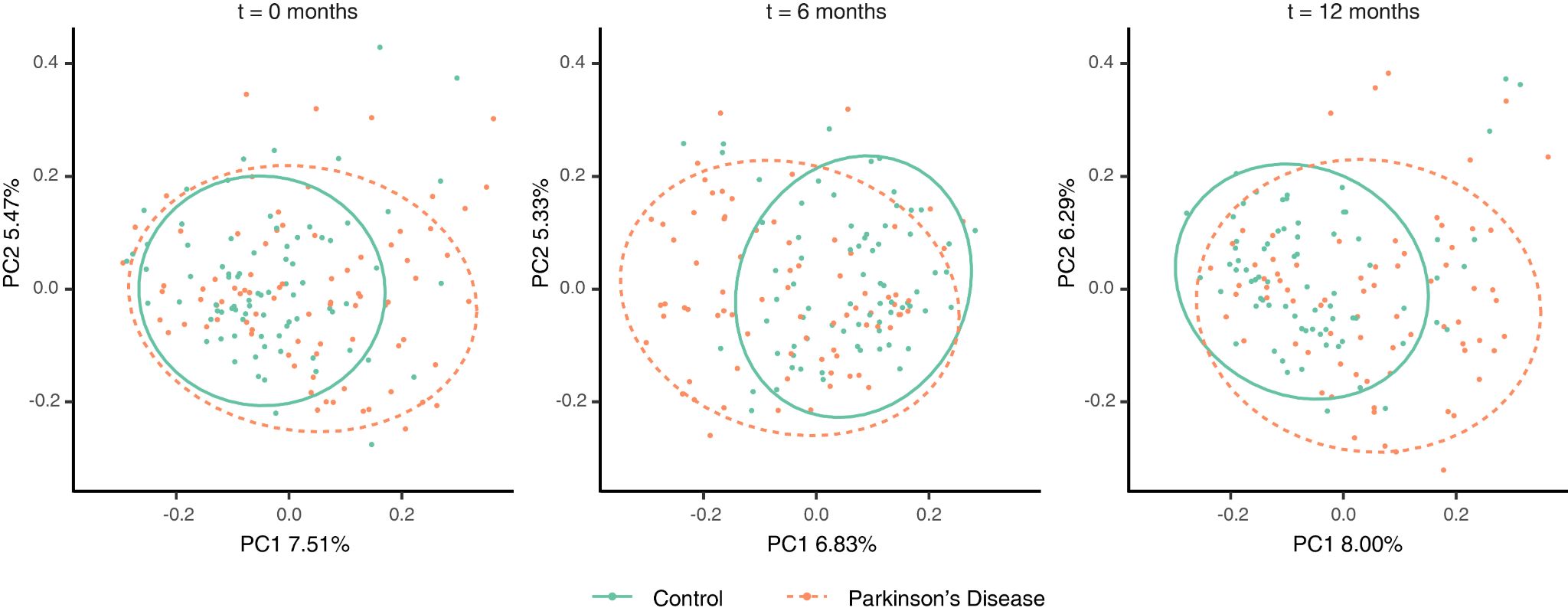


**Supplementary Figure 4: Comparable beta-diversity between the Parkinson’s disease (PD) and household control (HC) groups across sampling intervals.**Beta-diversity (bacterial richness between samples) explored by Principal Component Analysis (PCoA) with Bray-Curtis ordination at the Amplicon Sequencing Variant (ASV) level between the n=74 PD and n=74 HC groups across three, 0, 6 and 12 month time intervals for each participant. Overall, a small yet statistically significant difference in beta diversity was noted between the two groups (PERMANOVA p<0.01).

**
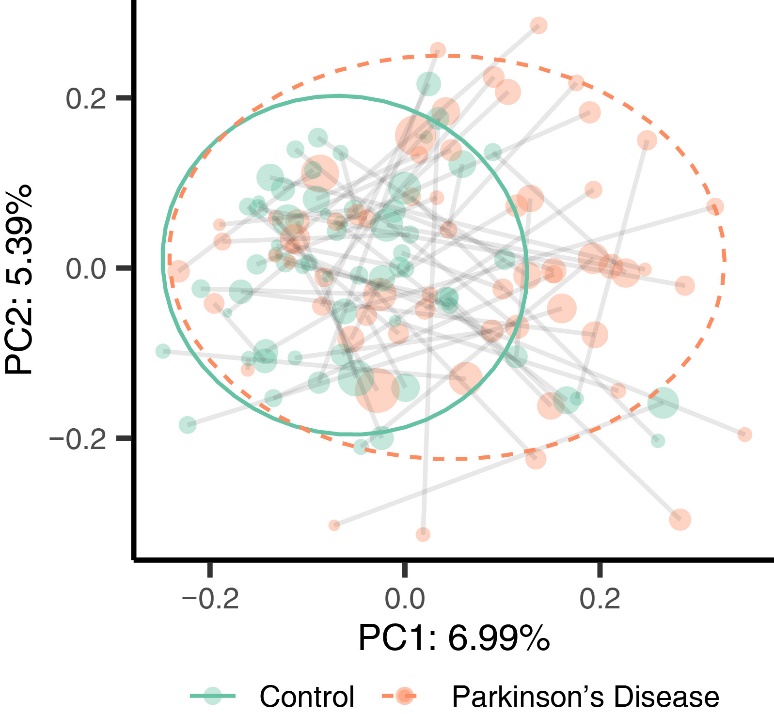
**

**Supplementary Figure 5: Cohabitant effect upon Beta-diversity between the Parkinson’s disease (PD) and household control (HC) groups across across sampling intervals.**Geographic / household matched HCs (n=74) and PD (n=74) patients were compared by Principal Coordinate Analysis with Bray-Curtis dissimilarity at the Amplicon Specific Variant (ASV) level for t=0, 6, 12 months timepoints, and are presented as pooled samples, PD (orange) and HC (green). Comparison of the first two principal components revealed varied beta diversity (extent of species diversity difference between two environments) between the groups, suggestive of a disease-related effect on GM composition that defines a PD-related GM composition (PERMANOVA; p<0.01). No significant difference was noted in respect to the individual timepoints between the groups. The size of the coloured circles indicate the variation of each participant’s diversity across the three time intervals. Coloured ellipses (solid green line and dotted orange line) represent 90% confidence region and the proportion of total variance represented by a given principal component is labelled on the respective axis.
